# Comparative Genomic Insights into Ecophysiology of Neutrophilic, Microaerophilic Iron Oxidizing Bacteria

**DOI:** 10.3389/fmicb.2015.01265

**Published:** 2015-11-13

**Authors:** Shingo Kato, Moriya Ohkuma, Deborah H. Powell, Sean T. Krepski, Kenshiro Oshima, Masahira Hattori, Nicole Shapiro, Tanja Woyke, Clara S. Chan

**Affiliations:** ^1^Department of Geological Sciences, University of Delaware, NewarkDE, USA; ^2^Japan Collection of Microorganisms, RIKEN BioResource CenterTsukuba, Japan; ^3^Delaware Biotechnology Institute, University of Delaware, NewarkDE, USA; ^4^Center for Omics and Bioinformatics, Graduate School of Frontier Sciences, University of TokyoKashiwa, Japan; ^5^Department of Energy Joint Genome Institute, Walnut CreekCA, USA

**Keywords:** iron oxidation, iron-oxidizing bacteria, biomineralization, *Ferriphaselus*, Gallionellales

## Abstract

Neutrophilic microaerophilic iron-oxidizing bacteria (FeOB) are thought to play a significant role in cycling of carbon, iron and associated elements in both freshwater and marine iron-rich environments. However, the roles of the neutrophilic microaerophilic FeOB are still poorly understood due largely to the difficulty of cultivation and lack of functional gene markers. Here, we analyze the genomes of two freshwater neutrophilic microaerophilic stalk-forming FeOB, *Ferriphaselus amnicola* OYT1 and *Ferriphaselus* strain R-1. Phylogenetic analyses confirm that these are distinct species within Betaproteobacteria; we describe strain R-1 and propose the name *F. globulitus.* We compare the genomes to those of two freshwater Betaproteobacterial and three marine Zetaproteobacterial FeOB isolates in order to look for mechanisms common to all FeOB, or just stalk-forming FeOB. The OYT1 and R-1 genomes both contain homologs to *cyc2*, which encodes a protein that has been shown to oxidize Fe in the acidophilic FeOB, *Acidithiobacillus ferrooxidans.* This *c*-type cytochrome common to all seven microaerophilic FeOB isolates, strengthening the case for its common utility in the Fe oxidation pathway. In contrast, the OYT1 and R-1 genomes lack *mto* genes found in other freshwater FeOB. OYT1 and R-1 both have genes that suggest they can oxidize sulfur species. Both have the genes necessary to fix carbon by the Calvin–Benson–Basshom pathway, while only OYT1 has the genes necessary to fix nitrogen. The stalk-forming FeOB share *xag* genes that may help form the polysaccharide structure of stalks. Both OYT1 and R-1 make a novel biomineralization structure, short rod-shaped Fe oxyhydroxides much smaller than their stalks; these oxides are constantly shed, and may be a vector for C, P, and metal transport to downstream environments. Our results show that while different FeOB are adapted to particular niches, freshwater and marine FeOB likely share common mechanisms for Fe oxidation electron transport and biomineralization pathways.

## Introduction

Microorganisms have long been associated with Fe oxidation in the environment, but the extent of their contribution to Fe and other elemental cycles is still unknown. There is often a question about whether Fe(II) oxidation is driven by biotic or abiotic processes, especially in oxic environments. Studies of microaerophilic neutrophilic Fe-oxidizing bacteria (FeOB) have demonstrated that microbes can contribute to Fe(II) oxidation, especially at lower oxygen concentrations at which abiotic Fe(II) oxidation is slow enough for microbes to compete (Rentz et al., 2007; Druschel et al., 2008). At the interface where Fe(II)-rich fluids meet oxygenated waters, O_2_ is often low; this is where we find abundant freshwater Betaproteobacterial FeOB and marine Zetaproteobacterial FeOB. Although traditionally studied in Fe microbial mats at groundwater seeps and hydrothermal vents, FeOB are being discovered in a variety of environments including rhizospheres, terrestrial aquifers, coastal sediments, and oceanic crustal boreholes (Emerson et al., 2007, 2010, 2015; Orcutt et al., 2011; McAllister et al., 2015), suggesting that microbes contribute significantly to Fe oxidation in these environments. It is intriguing that the microaerophilic neutrophilic FeOB mostly fall into two distinct classes of Proteobacteria; this presents the opportunity to learn about Fe oxidation in both freshwater and marine environments by comparing the physiology and genomes of Betaproteobacterial and Zetaproteobacterial FeOB.

In order to learn more about the roles of FeOB in nature, we need to determine the genes involved in the Fe oxidation electron transport system, which would then allow us to track FeOB activity. Difficulties in culturing FeOB have resulted in relatively few isolates and correspondingly few genomes: five genomes of microaerophilic FeOB reported thus far, two freshwater Betaproteobacteria and three marine Zetaproteobacteria (Singer et al., 2011; Emerson et al., 2013; Field et al., 2015). Comparative genomics, along with some initial genetic and proteomic work has led to candidate Fe oxidases and hypothetical models of Fe oxidation pathways (Singer et al., 2011; Liu et al., 2012; Emerson et al., 2013; Barco et al., 2015). The cytochrome MtoA from the freshwater FeOB *Sideroxydans lithotrophicus* was found to oxidize Fe *in vitro* (Liu et al., 2012). However, *mtoA* is rare in FeOB genomes, found in only the freshwater Betaproteobacteria *S. lithotrophicus* and *Gallionella capsiferriformans*, but not the marine Zetaproteobacteria, suggesting that it may be specific to freshwater FeOB. A proteomic/genomic study of the marine Zetaproteobacterium *Mariprofundus ferrooxydans* demonstrated high expression of a cytochrome *c*, Cyc2_PV -1_, which shares homology with the outer membrane Fe oxidase of *Acidithiobacillus ferrooxidans*, an acidophilic FeOB (Castelle et al., 2008; Barco et al., 2015). This led to a proposed model for Fe oxidation by *M. ferrooxydans* in which Cyc2_PV -1_ is the Fe oxidase, also involving a periplasmic cytochrome Cyc1 and the alternative complex III (ACIII) Act genes. The *cyc2* and *act* genes are attractive as potential markers of Fe oxidation because they are present in all of the sequenced FeOB genomes and are phylogenetically related. Further comparative genomics is needed to see if these genes are common across neutrophilic microaerophilic FeOB. FeOB genome analysis will also give us insight into how Fe oxidation fuels N, S, and C metabolisms, thus linking Fe and other major elemental cycles.

The primary way that FeOB affect the environment is through their biominerals. At circumneutral pH, Fe oxidation results in Fe(III) oxyhydroxides, which adsorb and coprecipitate organics, phosphate, arsenic, and other metals (e.g., Ferris, 2005; Borch et al., 2010). These oxides take the form of twisted ribbon-like stalks, hollow tubular sheaths, and granular (sometimes called “amorphous”) oxides. These are often associated with polysaccharides, which may play a role in stalk morphology, as well as binding Fe(III) and removing it from the cell (Chan et al., 2009, 2011). Of the five currently available microaerophilic FeOB that have sequenced genomes, all of the stalk-formers are marine (*M. ferrooxydans* sp. PV-1, M34, and EKF-M39) (**Table 1**). Strikingly, freshwater FeOB *Gallionella ferruginea* and *Ferriphaselus* sp. also produce a very similar twisted stalk (Vatter and Wolfe, 1956; Krepski et al., 2012; Kato et al., 2014), suggesting that the stalk plays an important role in both freshwater and marine Fe oxidation. However, we are still learning about stalk function; it must not be completely necessary since many FeOB (e.g., *G. capsiferriformans* and *S. lithotrophicus*) lack stalks (Emerson and Moyer, 1997; Weiss et al., 2007), and stalk-formers themselves do not always form stalks (Hallbeck and Pedersen, 1995). To understand FeOB physiology and contributions to environmental geochemistry, we need better insight into stalk formation and how it differs from other oxide morphologies.

**Table 1:** Overview of freshwater and marine neutrophilic microaerophilic FeOB genomes.

	Freshwater FeOB (Betaproteobacteria)				Marine FeOB (Zetaproteobacteria)		
	Stalk-formers		Non-stalk-formers		Stalk-formers		
	*Ferriphaselus amnicola* OYT1	*Ferriphaselus* sp. R-1	*Sideroxydans lithotrophicus* ES-1	*Gallionella capsiferriformans* ES-2	*Mariprofundus ferrooxydans* PV-1	*Mariprofundus* sp. M34	*Mariprofundus* sp. EKF-M39
Status	Draft	Draft	Complete	Complete	Draft	Draft	Draft
# of Contigs	23	25	1	1	32	36	45
Megabase pairs	2.68	2.44	3.00	3.16	2.87	2.74	2.72
GC content (%)	55.9	60.7	57.5	52.8	54.0	53.9	51.9
# of protein-coding regions	2639	2361	3049	3006	2920	2733	2715
# of tRNA	45	50	44	51	48	44	43
Reference	This study	This study	Emerson et al., 2013	Emerson et al., 2013	Singer et al., 2011	Field et al., 2015	Field et al., 2015

To better understand FeOB roles and mechanisms, we sequenced and analyzed the genomes of two freshwater stalk-forming FeOB, *Ferriphaselus amnicola* OYT1 and *Ferriphaselus* sp. R-1, within the order Gallionellales. We compare the OYT1 and R-1 genomes to other microaerophilic neutrophilic FeOB genomes to detect genes in common between (1) all seven aerobic FeOB genomes, focusing on electron carriers and Fe, C, N, and S metabolism, and (2) only the stalk-forming organisms OYT1, R-1, and *Mariprofundus* sp. We use electron microscopy to find novel Fe oxyhydroxide structures, which are much smaller than stalks. We suggest potential genes involved in each biomineral structure and discuss implications for the stalk’s purpose, the mobility of biogenic oxides, and FeOB effects on Fe cycling.

## Materials and Methods

### DNA Extraction, Sequencing, Assembly and Annotation

Genomic DNA of OYT1 and R-1 was extracted using a FastDNA spin kit for soil and the FastPrep instrument (MP Biomedicals) or a PowerSoil DNA isolation kit (MO-BIO) as previously described (Krepski et al., 2012; Kato et al., 2014). Whole-genome sequencing for OYT1 was performed using an Ion Torrent PGM system (Life Technologies) and 454 pyrosequencing (Roche) at the Center for Omics and Bioinformatics of the University of Tokyo, Japan. The generated reads were assembled using Newbler version 2.8 into 23 contigs with an N50 length of 423,423 bases. The draft genome sequences of OYT1 were annotated using Prokka (Seemann, 2014) that includes Prodigal (Hyatt et al., 2010), Aragorn (Laslett and Canback, 2004), Infernal (Nawrocki and Eddy, 2013) and RNAmmer (Lagesen et al., 2007) for detection of CDS, tRNA, non-coding RNA and rRNA, respectively. Whole-genome sequencing for R-1 was performed at the DOE Joint Genome Institute (JGI), USA, using the Illumina technology (Bennett, 2004). An Illumina Std shotgun library was constructed and sequenced on the Illumina HiSeq 2000 platform. Raw Illumina sequence data was artifact filtered and assembled via a hybrid approach using Velvet version 1.2.07 (Zerbino and Birney, 2008) and Allpaths-LG version r46652 (Gnerre et al., 2011). The final draft assembly of R-1 contained 25 contigs with an N50 length of 191,290 bases. The draft genome was annotated and integrated into the integrated microbial genomes (IMG) platform developed by the JGI (Markowitz et al., 2014).

### Sequence Analyses

To determine the closest sequences (up to 10 sequences with e-value <0.01) to all the predicted protein-coding regions (CDS) of each of the two strains, BLASTP (Altschul et al., 1997) was performed against the non-redundant protein database collected from the NCBI website. Based on the BLASTP result, the top hits of each CDS was taxonomically classified using MEGAN (Huson et al., 2007). Usearch (Edgar, 2010) was used for clustering CDS at 50% identity to determine whether the CDS is shared among genomes or not. Mauve (Darling et al., 2010) was used for comparison and visualization of the gene order in the genomes among OYT1, R-1 and other iron-oxidizers. The prediction of metabolic pathways was performed using the Kyoto Encyclopedia of Genes and Genomes (KEGG) pathway tool (Ogata et al., 1999). PSORTb (Yu et al., 2010) was used to predict subcellular localization of proteins. Functional properties of proteins coded by genes were predicted using InterProScan (Zdobnov and Apweiler, 2001). Putative *c*-type cytochrome (Cyc) proteins were found with the InterProScan ID, IPR009056 (Cytochrome c-like domain).

To construct phylogenetic trees, sequences related to a target gene or protein sequence were searched using BLAST (Altschul et al., 1997) or HMMER (Finn et al., 2011), and collected from the databases. The collected sequences with the target sequences were aligned using MUSCLE (Edgar, 2004) or MAFFT (Katoh and Standley, 2013). Gap positions were removed from the alignments using Gblock (Castresana, 2000). Phylogenetic trees were constructed by PhyML (Guindon et al., 2010) with the LG + I + G model or Fasttree (Price et al., 2010) with the JTT + CAT model.

The average nucleotide identity (ANI) between the two strains were calculated using JSpecies (Richter and Rosselló-Móra, 2009). The value of *in silico* DNA–DNA hybridization between the two strains was determined using GGDC 2.0 (Meier-Kolthoff et al., 2013).

### Time-lapse Imaging with Microslide Growth Chamber

Time-lapse experiments of OYT1 and R-1 were performed in microslide chambers as previously described by Krepski et al. (2013) with minor modifications. The modified Wolfe’s minimal medium (MWMM) buffered with MES (adjusted to pH 6.2) was used as a basal medium as previously described (Kato et al., 2014). An FeS plug (without agar) was used as an iron source. Differential interference contrast images were captured every 5 min at 400x total magnification using a Zeiss AxioImager Z1 light microscope (Zeiss, Oberkochen, Germany) equipped with a Zeiss Axiocam Mrm camera. A Zeiss AxioVision software was used for image processing.

### Fatty Acid Analysis, GC Content and Growth Test

The MWMM buffered with MES (adjusted to pH 6.2) was used as a basal medium to cultivate OYT1 an R-1 as previously described (Kato et al., 2014). FeS plug solidified with agar was used for an iron source. Fatty acid methyl esters and DNA base composition of R-1 were determined using the Microbial Identification System and HPLC method, respectively, as described (Kato et al., 2014).

For sulfide oxidation test, the basal medium solidified with low-melt-agarose (final 0.15% w/v) with Na_2_S plug (10, 1, 0.1, 0.01 or 0.001 mM) instead of FeS was used for cultivation. For the N_2_ fixation test, the basal medium with no nitrogen source (i.e., no ammonium and nitrate) and a mixture of N_2_/CO_2_/O_2_ gasses (95:4:1) in headspace was used for cultivation. A blank test with the mixture of Ar/CO_2_/O_2_ gasses in headspace was also performed. At least three transfers were conducted for the cultivation tests.

### Electron Microscopy

Scanning electron microscopy (SEM) and transmission electron microscopy (TEM) for OYT1 and R-1 cultures were performed at the Delaware Biotechnology Institute (DBI) Bioimaging Center of University of Delaware, USA, as previously described (Kato et al., 2013). In brief, samples for SEM were mounted on a 0.2-μm-pore-size polycarbonate filter, rinsed with ultrapure water, air dried, and coated with gold/palladium or carbon. Energy dispersive X-ray (EDX) spectrometry equipped with the SEM was used to detect elements for the samples. Samples for TEM were mounted on a lacey-carbon-coated copper grid, washed with ultrapure water, air dried, and coated with carbon. OYT1 and R-1 were cultured in the basal medium with FeS plug at 22°C for 18 h.

### Nucleotide Sequence Accession Numbers

The draft genome sequences of *Ferriphaselus* strains OYT1 and R-1 have been deposited in DDBJ/EMBL/GenBank under the accession numbers BBTH01000001-BBTH01000023 and JQKP01000001-JQKP01000025, respectively.

## Results and Discussion

### Genomic Features and Phylogeny of OYT1 and R-1

An overview of the genome sequences of two freshwater neutrophilic microaerophilic stalk-forming FeOB, *Ferriphaselus amnicola* strain OYT1 (Kato et al., 2014) and *Ferriphaselus* sp. strain R-1 (Krepski et al., 2012), is shown in **Table 1**, along with freshwater non-stalk-forming and marine stalk-forming FeOB strains (Singer et al., 2011; Emerson et al., 2013; Field et al., 2015). All of the 139 conserved single copy genes among bacterial genomes (Rinke et al., 2013) were found in each draft genome of OYT1 and R-1, indicating that both draft genomes are nearly complete.

**FIGURE 1:**
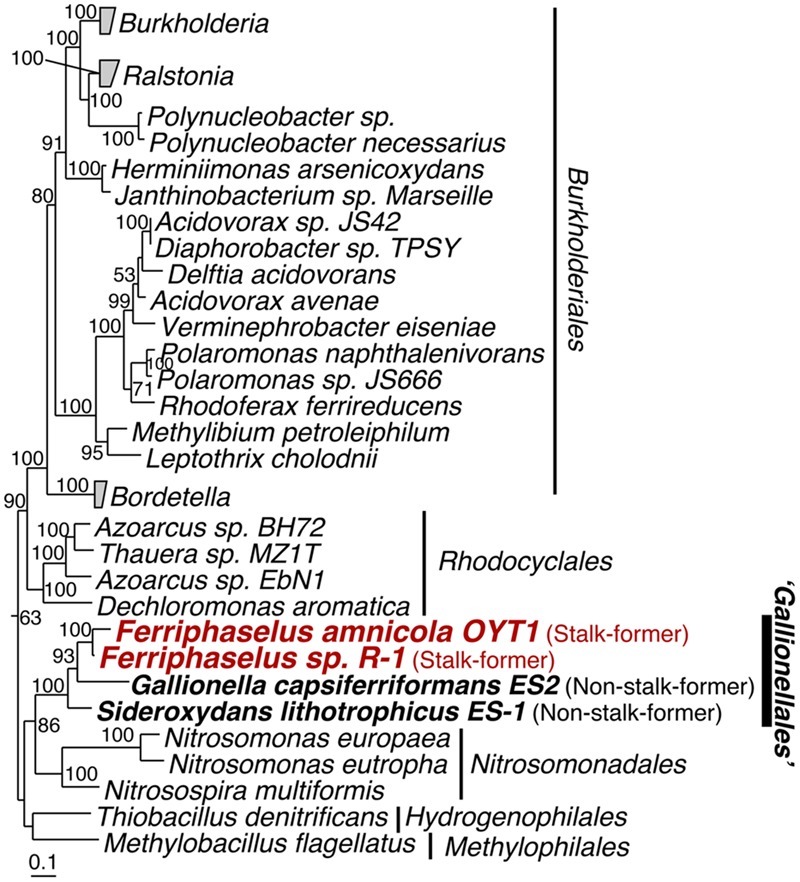
**Phylogenetic tree of concatenated amino acid sequences of 14 ribosomal proteins (RplA, RplB, RplC, RplD, RplE, RplF, RplK, RplM, RplN, RpsB, RpsC, RpsI, RpsJ and RpsS).** Bootstrap values (>50 of 100 replicates) are shown at the branch points. The concatenated amino acid sequence of these proteins of *Escherichia coli* K12 was used as the out-group (not shown).

Our phylogenetic analysis of concatenated ribosomal proteins, shows that the OYT1 and R-1 are clustered with ES-1 and ES-2 in the order *Gallionellales* within the Betaproteobacteria (**Figure 1**), confirming previous phylogenetic analyses of 16S rRNA genes (Krepski et al., 2012; Kato et al., 2013). Various analyses suggest that OYT1 and R-1 are separate species within the same genus. The 16S rRNA gene similarity between OYT1 and R-1 (97.8%) is lower than 98.65% of a species definition level (Kim et al., 2014). The ANI of the OYT1 and R-1 genomes (81.5%) is much lower than 95–96% of a species definition level (Kim et al., 2014). The value of *in silico* DNA–DNA hybridization between the two strains was 25.10 ± 2.40%, which is much lower than the 70% species level definition. Differences in major cellular fatty acids (Supplementary Table S1) and other phenotypic differences (see species description below) also support R-1 as a novel species, distinct from OYT1. Based on the previous work (Krepski et al., 2012; Kato et al., 2013), as well as the phylogenetic and taxonomic analyses presented here, we propose *Ferriphaselus globulitus* sp. nov., for strain R-1 as a novel species in the genus *Ferriphaselus*.

**FIGURE 2:**
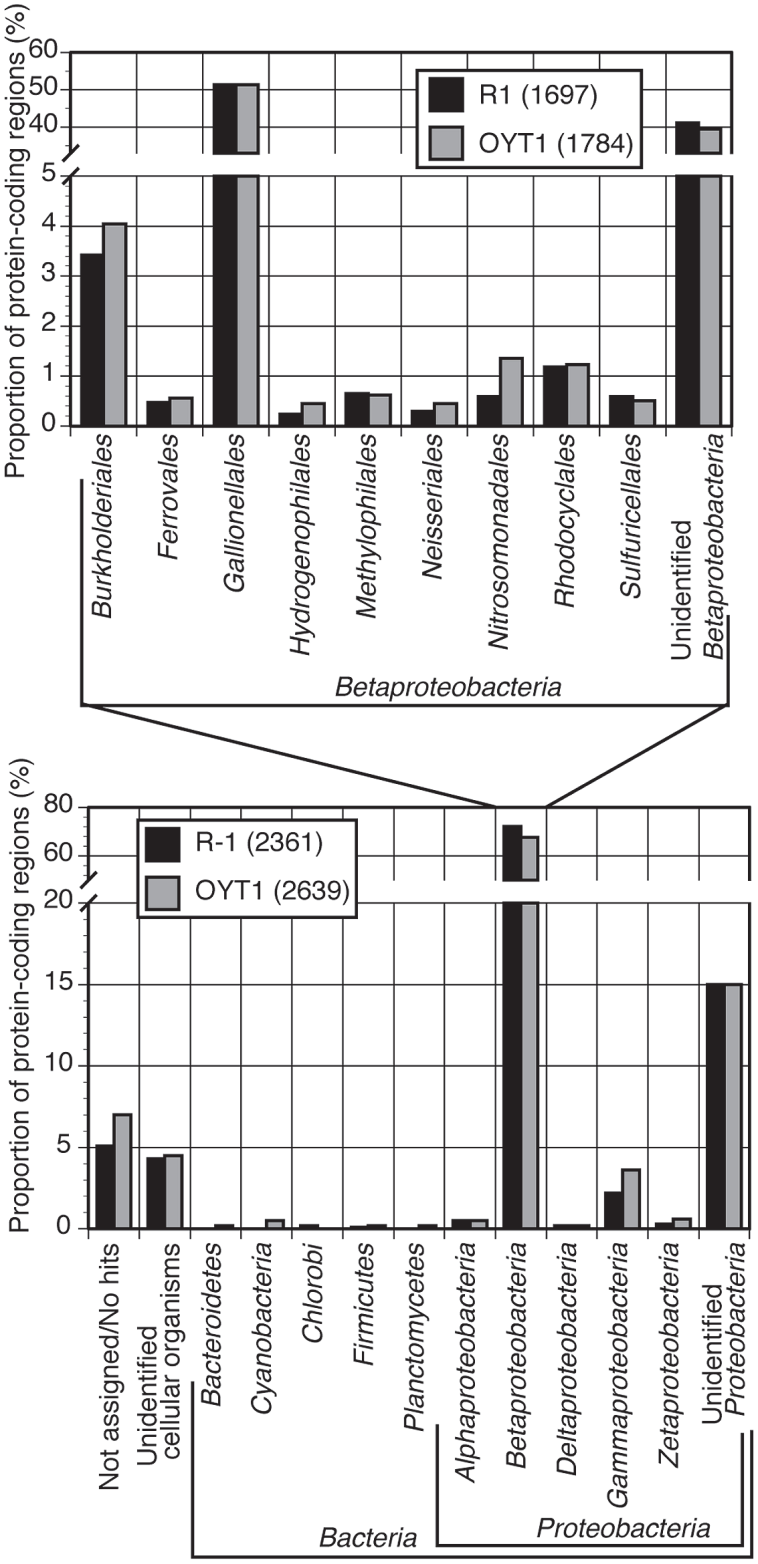
**Taxonomic classification of top hits of all CDS (genes) for OYT1 and R-1.** Proportion of protein-coding regions classified into each taxon to the all **(lower panel)** or betaproteobacterial CDS **(upper panel)** are shown. The total numbers of CDS are 2639 for OYT1 and 2361 for R-1.

Almost all of the OYT1 and R-1 genes were classified into Proteobacterial classes, with over 60% of the total genes were classified into Betaproteobacteria, and over half of these classified into Gallionellales (**Figure 2**). In order to look for commonalities between all microaerophilic neutrophilic FeOB, we noted 21 genes that were present in all seven genomes and closely related to each other (i.e., found in top 10 hits by BLAST search; Supplementary Table S2). Of these, 15 are monophyletic, which is remarkable since some genes are from Betaproteobacteria and others belong to Zetaproteobacteria, suggesting an evolutionary relationship (i.e., horizontal transfer). Thirty-seven genes were closely related between freshwater and marine stalk formers, but not to non-stalk-formers; four of them are monophyletic. Below, we analyze some of these genes in more detail (**Table 2**), in the context of Fe oxidation-related electron transport and Fe oxide stalk formation. In addition, OYT1 and R-1 shared 373 genes with each other, but not the other five microaerophilic FeOB (Supplementary Table S3); some of them may be related to the novel biomineral morphology that we describe below.

**Table 2:** Temporary identification for the 20 CDS focused in this study.

ID	OYT1 locus_tag	R-1 locus_tag	Potential role	Gene name	Monophyly^∗^
CDS1	OYT1_00168	DM08DRAFT_00346	Iron oxidation	*cyc2*	×
CDS2	OYT1_00085	DM08DRAFT_02259	Iron oxidation	*actA*	∘
CDS3	OYT1_00084	DM08DRAFT_02260	Iron oxidation	*actB1*	∘
CDS4	OYT1_00083	DM08DRAFT_02261	Iron oxidation	*actB2*	∘
CDS5	OYT1_00082	DM08DRAFT_02262	Iron oxidation	*actC*	∘
CDS6	OYT1_00081	DM08DRAFT_02263	Iron oxidation	*actD*	∘
CDS7	OYT1_00080	DM08DRAFT_02264	Iron oxidation	*actE*	∘
CDS8	OYT1_00079	DM08DRAFT_02265	Iron oxidation	*actF*	∘
CDS9	OYT1_00167	DM08DRAFT_00347	Stalk formation	*bcsB*	∘
CDS10	OYT1_00166	DM08DRAFT_00348	Stalk formation	*xagB*	∘
CDS11	OYT1_00165	DM08DRAFT_00349	Stalk formation	*xagC*	∘
CDS12	OYT1_00164	DM08DRAFT_00350	Unknown	*xagD*	∘
CDS13	OYT1_00976	DM08DRAFT_01732	Dread formation	*bcsA*	–
CDS14	OYT1_00975	DM08DRAFT_01733	Dread formation	*bcsB*	–
CDS15	OYT1_00974	DM08DRAFT_01734	Dread formation	*bcsZ*	–
CDS16	OYT1_00296	DM08DRAFT_00247	Dread formation	*bcsC*	–
CDS17	OYT1_01454	DM08DRAFT_00108	Cell wall synthesis	*mltA*	∘
CDS18	OYT1_01534	DM08DRAFT_01856	Cell wall synthesis	*ampG*	∘
CDS19	OYT1_02351	DM08DRAFT_02295	Unknown	–	∘
CDS20	OYT1_02621	DM08DRAFT_02432	Energy generation	*sthA*	∘

*^∗^ ∘, monophyletic clustering for FeOB; ×, non-monophyletic clustering for FeOB; –, found in only OYT1 and R-1.*

### Catabolism and Anabolism

#### Iron Oxidation and Electron Transport

The OYT1 and R-1 genomes both lack *mtoA* and *mtoB*, suggesting that these may be rare, and not part of a common mechanism of Fe oxidation amongst freshwater FeOB (**Table 3**). However, both genomes include homologs of *cyc2*_PV -1_, a gene found in *M. ferrooxydans* PV-1 that encodes a Cyc2-like protein, which Barco et al. (2015) proposed as a candidate iron-oxidizing protein, based on high protein expression. Predicted to be an outer membrane *c*-type cytochrome, Cyc2_PV -1_ has homologs encoded in all of the other neutrophilic microaerophilic FeOB genomes, as well as metagenomes containing *Gallionellales* and the *Zetaproteobacteria* (Barco et al., 2015). The presence in OYT1 and R-1 supports the hypothesis that this gene is required by microaerophilic FeOB, including both Beta- and Zetaproteobacteria.

**Table 3:** List of proteins potentially involved in iron oxidation pathway of the neutrophilic microaerophilic FeOB.

	Freshwater FeOB (Betaproteobacteria)				Marine FeOB (Zetaproteobacteria)			
	Stalk-formers		Non-stalk-formers		Stalk-formers			unknown
	*Ferriphaselus*		*Sideroxydans*	*Gallionella*	*Mariprofundus*			–
Proteins	OYT1	R-1	ES-1	ES-2	PV-1	M34	EKF-M39	Zeta SAGs (24)^1^
Cyc2	+^2^	+	+	+	+	+	+	9
MtoA	N.F.^3^	N.F.	+	+	N.F.	N.F.	N.F.	N.F.
Cyc1	N.F.	N.F.	+	-^4^	+	+	+	12
Alternative Complex III	+	+	+	+	+	+	N.F.	3
Cytochrome bc1 complex	+	+	+	-	+	+	+	10
Cytochrome bd quinol oxidase	+	N.F.	+	+	+	+	+	4
Cbb3-type cytochrome c oxidase	+	+	+	+	+	+	+	14
NADH dehydrogenase	+	+	+	+	+	+	+	19
Reference	This study	This study	Emerson et al., 2013	Emerson et al., 2013	Singer et al., 2011	Field et al., 2015	Field et al., 2015	Field et al., 2015

*^1^Numbers found in the total 24 single amplified genomes (SAGs) are indicated; ^2^present; ^3^not found in the incomplete genomes; ^4^absent in the complete genome.*

**FIGURE 3:**
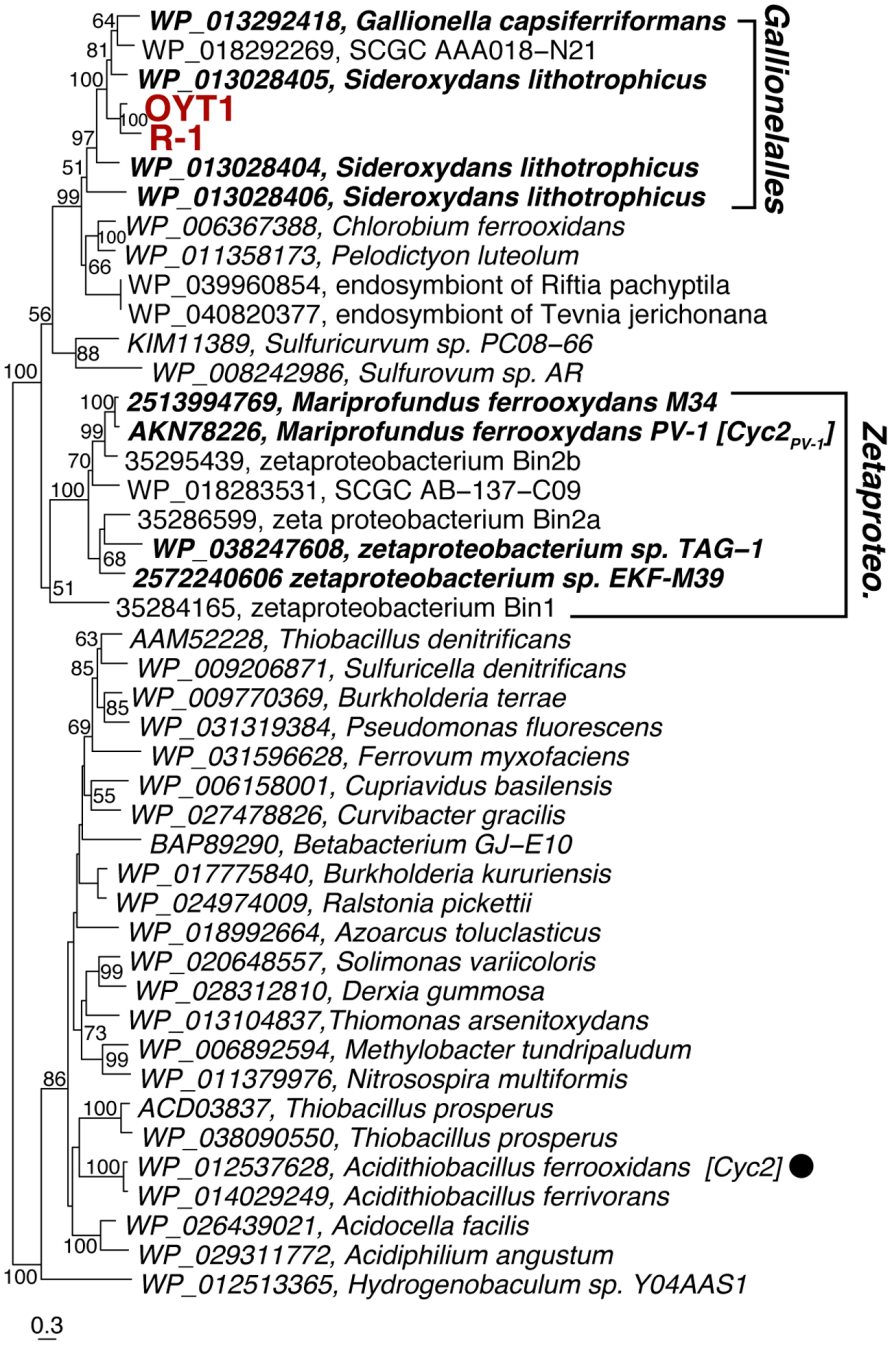
**Phylogenetic tree of Cyc2_PV -1_ and its homologs corresponding to CDS1.** The tree was constructed using an alignment dataset of the whole protein sequences without gap positions. Bootstrap values (>50 of 100 replicates) are shown at the branch points. Betaproteobacterial and Zetaproteobacterial neutrophilic FeOB are shown in bold. The filled circle following the species name indicates that the function of the protein has been identified.

In the OYT1 and R-1 genomes, the homologs of *cyc2*_PV -1_ (CDS1) were not originally annotated as cytochromes due to the low similarity to previously known cytochromes; however, both contain a Cys-X-X-Cys-His heme-binding motif near the N terminus, as in PV-1. Cyc2_PV -1_ homologs in neutrophilic chemolithotrophic FeOB generally cluster together, distinct from the Cyc2 of *A. ferrooxidans*, with Zetaproteobacteria in a separate grouping from the freshwater Gallionellales (**Figure 3**). However, the cluster of neutrophilic chemolithotrophic FeOB Cyc2 sequences also include several homologs from other organisms, including an iron-oxidizing phototroph *Chlorobium ferrooxidans*, as well as two sulfur-oxidizing species and two marine endosymbionts. The similarity to *C. ferrooxidans* and *A. ferrooxidans cyc2* suggests that this could be a common Fe oxidation mechanism across different types of Fe-oxidizing bacteria.

While OYT1 and R-1 share many other electron transport genes with PV-1 and other microaerophilic FeOB (see below), we did not find any homologs of *cyc1*_PV -1_, the gene that encodes a periplasmic cytochrome, which was also implicated in Fe oxidation by its high expression in the PV-1 proteome (Barco et al., 2015); it is also missing in the genome of *Gallionella* ES-2, suggesting Cyc1 is not a common periplasmic electron carrier amongst FeOB. However, 16 genes encoding putative *c*-type cytochromes were found in the OYT1 genome, and 11 were found in the R-1 genome, in addition to the *cyc2* homolog (Supplementary Table S4); some of these Cyc (eight for OYT1, five for R-1) could serve as periplasmic electron carriers, substituting for Cyc1.

**FIGURE 4:**
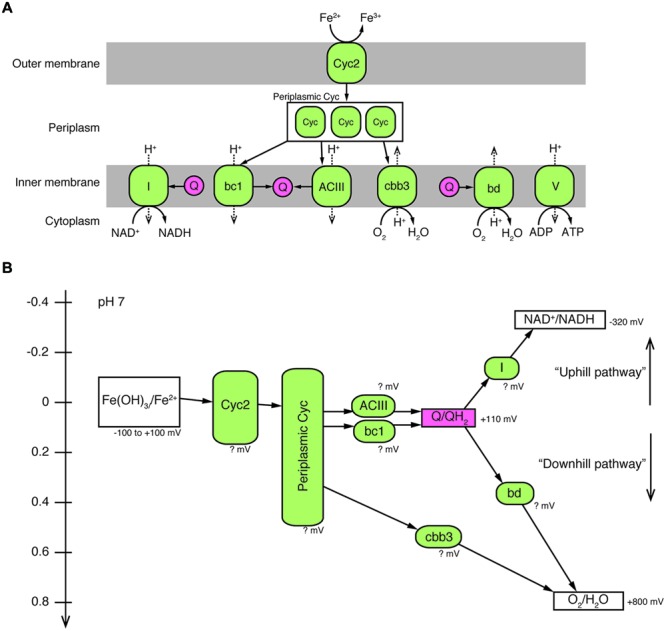
**A proposed model of electron pathway from Fe^2+^ to O_2_ and generation of NADH and ATP in *Ferriphaselus* OYT1 and R-1. (A)** Predicted locations of proteins (colored in green) potentially involved in the electron pathway. **(B)** Redox potentials of each reaction in the electron pathway. It should be noted that the redox potentials of all proteins of the FeOB are still unclear, and that the cytochrome *bd* complex was not found in the R-1 genome. I, Complex I (NADH dehydrogenase); bc1, cytochrome *bc*_1_ complex; ACIII, alternative complex III; Q, quinone pool (colored in pink); Cyc, cytochrome *c*; bd, cytochrome *bd* complex; cbb3, *cbb*_3_-type cytochrome *c* oxidase; V, Complex V (ATPase).

Once electrons are passed from the outer membrane electron carriers to periplasmic Cyc, some of the electrons must be used to produce NADH by reverse electron transport. Based on genes present in their genomes, OYT1 and R-1 can pass electrons to the cytochrome *bc*_1_ complex (Complex III), which reduces a quinone, which in turn reduces NAD+ to NADH via the NADH dehydrogenase (Complex I) (**Figure 4**); this is similar to the model for all of the other sequenced microaerophilic FeOB as well as the acidophilic FeOB, *A. ferrooxidans* (Brasseur et al., 2002). Instead of Complex III, it is possible that OYT1 and R-1 use the alternative complex (AC) III to pass electrons to quinones. Like all other microaerophilic FeOB, OYT1 and R-1 genomes contain a gene cluster that show high similarity to *act* genes of ACIII (Yanyushin et al., 2005). ACIII and the related Qrc function as quinol oxidases or quinone reductases, as characterized in *Chloroflexus aurantiacus, Rhodothermus marinus*, and *Desulfovibrio vulgaris* (Pereira et al., 2007; Gao et al., 2009, 2013; Refojo et al., 2010; Venceslau et al., 2010) and as proposed for an uncultivated *Chromatiaceae* (Wang et al., 2015). Given the few studies on ACIII, its placement in FeOB electron transport systems is highly speculative.

**FIGURE 5:**
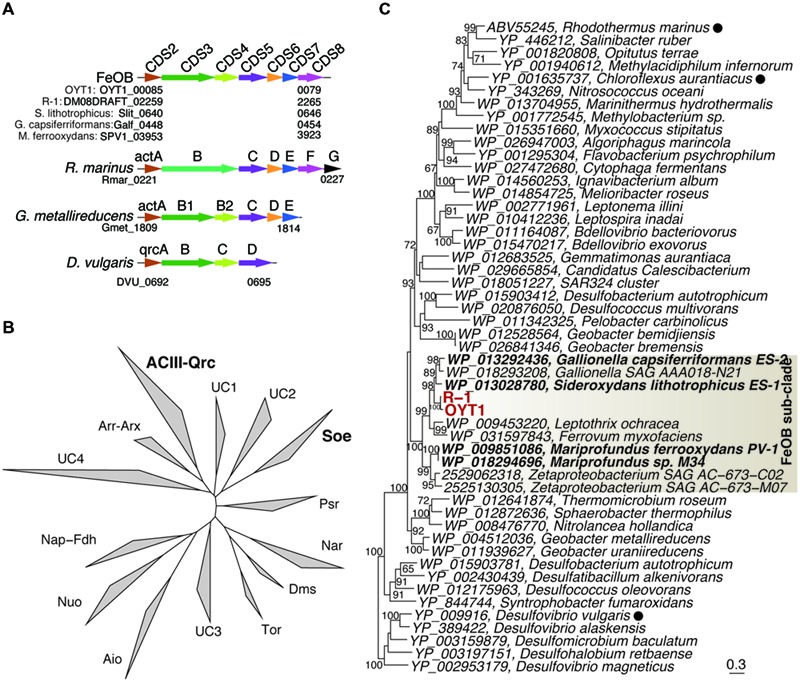
**The ACIII-like gene cluster. (A)** Orders of protein-coding regions related to genes in the ACIII-like gene cluster. Homologs among the genomes are shown in the same color. Cyc, *c*-type cytochrome. **(B)** Phylogenetic tree for proteins corresponding to ActB1 in the complex iron-sulfur molybdoenzyme super-family including ACIII, Qrc, Aio, arsenite oxidase; Arr, arsenate respiratory reductase; Arx, alternative arsenite oxidase; Nap, periplasmic nitrate reductases; Fdh, formate dehydrogenases; Nuo, NADH:ubiquinone oxidoreductase; Tor, Trimethylamine *N*-oxide reductase; Dms, dimethyl sulfoxide reductase; Nar, respiratory nitrate reductase; Psr, polysulfide reductases; Soe, sulfite-oxidizing enzymes. UC indicate unclassified clades. **(C)** Phylogenetic tree for ActB1 and ActB1-like proteins corresponding to CDS3 in the ACIII-Qrc clade. Bootstrap values (>50 of 100 replicates) are shown at the branch points. Filled circles following the species names indicate that the function of the proteins has been identified.

The *act* gene clusters in both OYT1 and R-1 genomes each contain seven genes (CDS2–8; **Table 2**) related to *actAB1B2CDEF* (**Figure 5A**); *actB* is split into two subunits unlike in *R. marinus*. The closest relatives of all seven genes based on a homology search are those of FeOB (Supplementary Table S2), in addition to two Betaproteobacterial FeOB, i.e., *Ferrovum myxofaciens* (Johnson et al., 2014) and *Leptothrix ochracea* (Fleming et al., 2011) (Genbank accession number: AJUC00000000). Notably, the OYT1 and R-1 *act* genes are in the same order as in other FeOB genomes, with *actB* split into two: *actB1* with the [4Fe–4S] binding motif (CX_2_CX_3_CX_27_C), which encodes a protein similar to a molybdopterin oxidoreductase, and *actB2*, which encodes a protein similar to an iron-sulfur protein, both within the complex iron-sulfur molybdoenzyme (CISM) super-family (**Figure 5B**) (Rothery et al., 2008). Each of the *act* genes of the Betaproteobacterial and Zetaproteobacterial FeOB isolates are clustered into a sub-clade within the ACIII-Qrc clade (*actB1*-like genes shown in **Figure 5C**; other *act* genes in Supplementary Figure S1). The phylogenetic analyses suggest a horizontal gene transfer between the Betaproteobacterial and Zetaproteobacterial FeOB.

Electrons not used in reverse (“uphill”) electron transport would be directed toward a terminal oxidase (“downhill”) in order to generate proton motive force (**Figure 4**). For OYT1, the terminal oxidase could either be the *cbb*_3_-type cytochrome *c* oxidase (OYT1_00427, 00429, 00430) or the cytochrome *bd* quinol:O_2_ oxidoreductase (OYT1_01855, 01856); the R-1 genome contained genes for the *cbb*_3_-type complex (DM08DRAFT_00513, 00515, 00516) but not the cytochrome bd oxidase. Both types of terminal oxidases have high affinity for O_2_ (Pitcher and Watmough, 2004; Borisov et al., 2011), consistent with the microaerophily of these FeOB. As expected, both OYT1 and R-1 genomes contain genes encoding an F-type ATPase (OYT1_01226–01233, DM08DRAFT_01947–01954) that converts the proton motive force to ATP.

#### Sulfur Oxidation

OYT1 and R-1 have thus far proven to be obligate Fe-oxidizers that do not grow by sulfide or thiosulfate oxidation (Krepski et al., 2012; Kato et al., 2014). The other FeOB isolates (*G. capsiferriformans* and *Mariprofundus* sp.) are also obligate Fe-oxidizers, except for *S. lithotrophicus*, which was shown to oxidize thiosulfate once genome analysis showed that it had *sox* genes needed for dissimilatory sulfur oxidation (Emerson et al., 2013). Given that culture tests have proven wrong in the past, we evaluated the OYT1 and R-1 genomes for sulfur metabolism genes, and also retested both strains for sulfide and thiosulfate oxidation. Although both strains failed to grow, genome analyses revealed genes involved in dissimilatory sulfur oxidation (*dsr, sqr, soe*) as described below, as well as sulfate assimilation (*cys*). Other genes related to sulfur oxidation (e.g., *sor, apr, sat*, and *sox*) were not found.

In both OYT1 and R-1 genomes, we found genes encoding sulfide:quinone oxidoreductase (Sqr), which oxidizes sulfide to elemental sulfur. Phylogenetic analysis indicates that the Sqr of OYT1 and R-1 is classified in Type I clade (Supplementary Figure S2; Marcia et al., 2010), along with other Betaproteobacterial SOB, including *Sideroxydans* ES-1. The Type I Sqr has affinity to sulfide at micromolar level, and plays a role in sulfide-dependent respiration, in addition to sulfide detoxification (Marcia et al., 2010).

Dissimilatory sulfite reductase (*dsr*) genes were found in the OYT1 and R-1 genomes (Supplementary Figure S3A), as well as in the *Sideroxydans* ES-1 genome (Emerson et al., 2013). Dsr catalyzes elemental sulfur oxidation to sulfite in some sulfur-oxidizing bacteria such as *Allochromatium vinosum* (Dahl et al., 2005) and, on the other hand, sulfite reduction to elemental sulfur in sulfate-reducing microorganisms (e.g., Wagner et al., 1998). The OYT1 and R-1 *dsr* gene clusters each contain 13 genes, *dsrABEFHCMKLJOPN*; as in ES-1, the cluster in the OYT1 is located near a sulfite oxidation *soe* gene cluster (Supplementary Figure S3A). DsrB of OYT1 and R-1 are closely related to *Sideroxydans* ES-1 and other Betaproteobacterial SOB (Supplementary Figure S4), suggesting that the Dsr complex plays a role in sulfur oxidation rather than sulfate reduction. In addition, a reverse DSR-associated gene cluster (rDAGC) was found upstream of the Dsr cluster in the OYT1 and R-1 genomes (Supplementary Figure S3A). The rDAGC has been found in the genomes of Betaproteobacterial SOB, including *Sideroxydans* ES-1 (Watanabe et al., 2014), and may be involved in the regulation of the *dsr* genes (Venceslau et al., 2014).

In the OYT1 and R-1 genomes, we found *soeABC* genes (Supplementary Figure S3A) encoding sulfite oxidizing enzyme (Soe), similar to that found in *A. vinosum* (Dahl et al., 2013). The Soe of OYT1 and R-1 cluster with other Soe within the CISM super-family (**Figure 5B**); this Soe cluster contains sulfide-, elemental sulfur-, and sulfite-oxidizing bacteria (SOB) including *Sideroxydans* ES-1 (Supplementary Figure S3B). In combination with Dsr, the Soe in OYT1 and R-1 may allow for elemental sulfur oxidation to sulfate, though S(0) oxidation has not yet been tested. Our findings suggest that further physiological experiments may confirm their ability to oxidize reduced sulfur species.

#### Carbon Fixation

Most microaerophilic FeOB isolated to date, including OYT1 and R-1, grow without an organic carbon source, so they are interpreted to be autotrophic (Kato et al., 2012; Krepski et al., 2012). Genomic analyses have supported this conclusion, as genes for carbon fixation via the Calvin–Benson–Basshom (CBB) pathway, including ribulose-1,5-bisphosphate carboxylase/oxygenase (RubisCO) genes are found in the genomes of *M. ferrooxydans* (Singer et al., 2011; Field et al., 2015) and *G. capsiferriformans* and *S. lithotrophicus* (Emerson et al., 2013), while *cbbM*-targeted PCR demonstrated the presence of RubisCO form II in OYT1 by PCR (Kato et al., 2013). Furthermore, RubisCO is significantly expressed in the *M. ferrooxydans* proteome (Barco et al., 2015). In this study, we found that both OYT1 and R-1 have all the genes necessary for the CBB pathway. A *cbbM* gene (indicative of RubisCO form II) is present in both OYT1 and R-1 genomes, while *cbbL* (indicative of RubisCO form I) is only found in R-1; there was no evidence of other carbon fixation pathways. The genes of RubisCO form II were found in all of the microaerophilic FeOB genomes; Form II is adapted to low O_2_ and high CO_2_ conditions (Badger and Bek, 2008), which is consistent with the microaerophilic lifestyle. RubisCO form I corresponds to higher O_2_ and low CO_2_ conditions (Badger and Bek, 2008); the FeOB that have both Form I and II (R-1, *S. lithotrophicus* ES-1, *M. ferrooxydans* PV-1 and M34) likely can survive in a wider range of O_2_ and CO_2_ conditions. Phylogeny of the FeOB CbbM and CbbL show that not all FeOB RubisCO are closely related (Supplementary Figure S5), suggesting a complex acquisition/evolutionary history.

#### Nitrogen Fixation

Thus far, there is genomic and physiological evidence of nitrogen fixation in some, though not all microaerophilic FeOB. The *nifH* gene has been also found in *Sideroxydans* ES-1 and *Mariprofundus* M34 and EKF-M39, but not *M. ferrooxydans* PV-1 or *G. capsiferriformans* ES-2. ES-1 can grow with N_2_ as the sole N source, though it is the only one of those strains that has been tested. The OYT1 and R-1 genomes both possess *nif* genes for nitrogen fixation. The minimum set of *nif* genes (*nifHDKENB*) required for nitrogen fixation (Dos Santos et al., 2012) was found in the OYT1 genome, consistent with the cultivation result that OYT1 grew on N_2_ as the sole nitrogen source. In contrast, *nifHDK* genes were not found in the R-1 genome, and the growth of R-1 on N_2_ as the sole nitrogen source has not been observed in our tests. NifH (dinitrogenase reductase) is one of the key components of the nitrogenase enzyme complex and is a commonly used marker gene for nitrogen fixation. The NifH of the FeOB are all Type I, a group which contains aerobic and facultative anaerobes (Supplementary Figure S6) (Chien and Zinder, 1994). The presence of *nif* genes in several neutrophilic Fe-oxidizers, including OYT1, demonstrates a link between Fe oxidation and N_2_ fixation in the environment at circumneutral pH.

### Extracellular Structures

**FIGURE 6:**
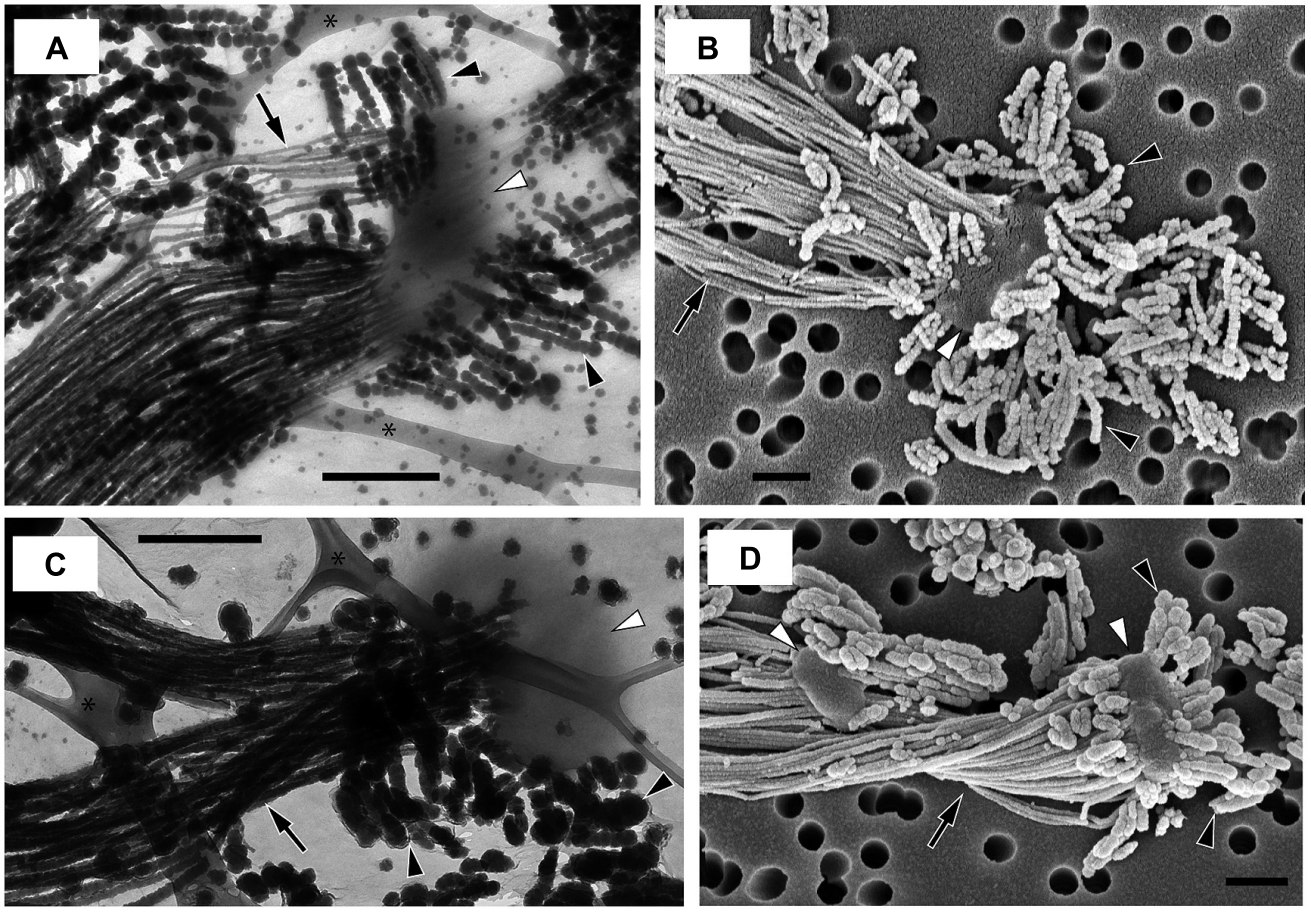
**Electron micrographs of strains OYT1 and R-1. (A,B)** OYT1 and **(C,D)** R-1. **(A,C)** TEM and **(B,D)** SEM images. **(A,C)** asterisk (^∗^) indicates lacey carbon on the TEM grids. White arrowheads, cells; black arrowheads, dreads; black arrows, stalks. Bars, 500 nm.

Many microorganisms have extracellular structures, such as pili and flagella, and secrete extracellular polymeric substances (EPS), that are involved in motility, attachment on surfaces, biofilm production and cell–cell interaction, and give the microorganisms competitive advantages (e.g., Mattick, 2002; Flemming and Wingender, 2010; Guttenplan and Kearns, 2013). FeOB stalks containing polysaccharides (Chan et al., 2009, 2011; Krepski et al., 2012; Kato et al., 2014) are one example of EPS. Furthermore, in this study, we found other extracellular structures that consisted of tens-nanometer-sized iron biominerals (nanoBIOS), morphologically distinct from the stalks, in OYT1 and R-1 cultures (**Figure 6**; Supplementary Figure S7). We call these structures “dreads” based on their morphology, which resembles the dreadlock hairstyle. Based on genomic analysis and microscopic observation, genes potentially involved in the formation of stalks and dreads and the ecophysiological roles of these structures are discussed.

#### Stalk Formation

Time-lapse imaging using microslide growth chambers showed that OYT1 and R-1 cells first attached to the glass surface, and then produced stalks (Video S1 for OYT1, data not shown for R-1). The average elongation rates were 1.4 (±0.4) μm/h and 1.2 (±0.6) μm/h for OYT1 and R-1 (*n* = 6 cells for each), respectively, which are similar to that of *Mariprofundus* PV-1 (2.2 μm/h; Chan et al., 2011).

**FIGURE 7:**
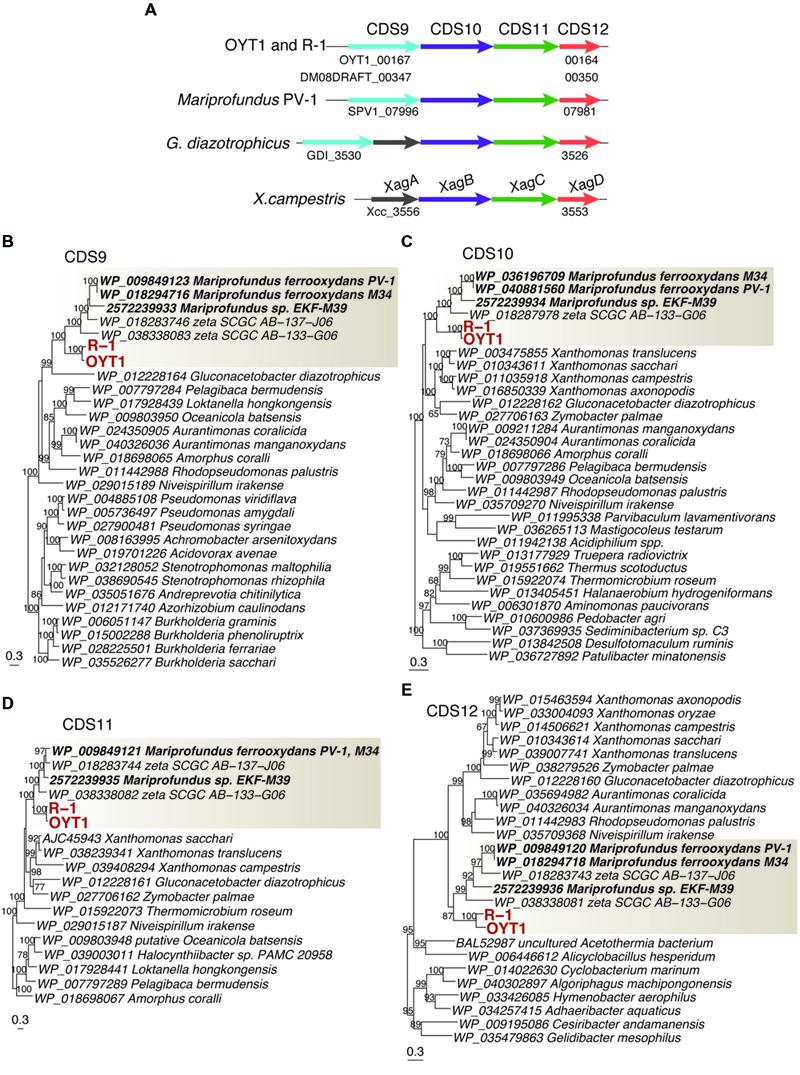
**The gene cluster containing *xag-*like genes. (A)** Gene order in the gene cluster. Homologs among the genomes are shown in the same color. **(B)** Phylogenetic trees for proteins related to **(B)** CDS9 (BcsB-like), **(C)** CDS10 (XagB-like), **(D)** CDS11 (XagC-like) and **(E)** CDS12 (XagD-like). Bootstrap values (>50 of 100 replicates) are shown at the branch points. The gray box indicates a clade including FeOB isolates and SAGs of Betaproteobacteria and Zetaproteobacteria.

We found a cluster of four genes (CDS9–12; **Figure 7A**; **Table 2**, Supplementary Table S2) that was only present in the stalk forming FeOB (*Mariprofundus* and *Ferriphaselus* strains). Our phylogenetic analysis shows that each of CDS9–12 in the stalk forming FeOB is clustered into a distinct clade from the others, and that freshwater FeOB are clearly separated from those of the marine FeOB (**Figures 7B–E**). These results suggest that the genes were horizontally transferred between the common ancestor of the *Mariprofundus* and that of the *Ferriphaselus*.

CDS9 is annotated as a cellulose synthase regulator protein, classified in BcsB family (pfam ID: PF03170) that is involved in cellulose synthesis (Römling, 2002). CDS10–12 are similar to *xagBCD* genes, which are involved in extracellular polysaccharide production and biofilm formation in *Xanthomonas campestris* (Tao et al., 2010). The *xagB*-like CDS10 was annotated as beta-monoglucosyldiacylglycerol synthase, one of the glucosyltransferases that is needed to synthesize polysaccharides (Lairson et al., 2008). No CDS with considerable similarity to *xagA* gene was found in the OYT1 and R-1 genomes. Considering the monophyletic clustering of the genes of the stalk forming FeOB and the relatedness to extracellular polysaccharide production, the gene cluster of the CDS9–12 are good candidates for involvement in FeOB stalk production.

We found other genes related to polysaccharide synthesis in the OYT1 and R-1 genomes, which have already been reported in the other microaerophilic FeOB (Singer et al., 2011; Emerson et al., 2013; Bennett et al., 2014). Further studies are needed to reveal whether the cluster is related to the synthesis of extracellular polysaccharide or lipopolysaccharide of an outer membrane component.

#### Dreads Formation

We discovered new extracellular structures using a combination of light and electron microscopy of OYT1 and R-1 cultures. The time-lapse images (Supplementary Video S1) show that OYT1 cells produce amorphous extracellular structures distinct from stalks, although their morphology is unclear by the light microscopic images. The electron microscopy indicates that the amorphous structures are clusters of dreadlock-shaped minerals (“dreads”) and that there are cells attached to the dreads, in addition to the stalk structure (**Figure 6**). These results provide evidence that the cells excrete dreads. The dreads extended from all sides of a cell, whereas the stalks were produced from only one side of the cell. The widths of dreads are larger and more varied than that of stalks in 1-day old cultures: stalks and dreads of OYT1 are 23 (± 6) nm and 63 (±16) nm wide, respectively; those of R-1 are 39 (±10) nm and 93 (±23) nm, respectively (*n* ≥ 20 dreads or stalks of different cells). Dreads appeared segmented, especially in the OYT1 cultures (**Figure 6**; Supplementary Figure S7). The diameter of dread segments for both strains tends to be smaller (<30 nm, in some cases) nearer the cells, which may reflect the degree of mineralization. Dreads are typically <500 nm in length, clearly much shorter than that of stalks (>100 μm in many cases). SEM-EDX analysis shows that the dreads, as well as the stalks, of OYT1 and R-1 contain Fe and P (Supplementary Figure S8), likely representing phosphate adsorbed onto Fe oxyhydroxides. In the case of the stalks, this is consistent with the previous reports of the stalks of cultured and environmental *Gallionella* sp. and *Mariprofundus* PV-1 (Chan et al., 2009, 2011; Suzuki et al., 2011).

Dread-like structures have not previously been reported in other neutrophilic microaerophilic FeOB; however, it is possible that they have been overlooked. On re-examining electron micrographs in the literature, we found dread-like precipitates associated with a stalk-forming *Gallionella ferruginea* (Figures 1 and 6 shown in Vatter and Wolfe, 1956). However, the authors thought that segmented, dread-like precipitates were ferrous sulfides used as the iron source for cultivation. The authors also found other precipitates called “granules” on the cells (Figures 8–10 shown in Vatter and Wolfe, 1956) and discussed that they might have been secreted inside the cell and were related to segmented stalk strands, or else were abiotic precipitates sorbed to the cell surface. We only observed stalks and dreads on OYT1 and R-1 cells, but did not consistently see granules, so we can only confirm that dreads and stalks are distinct, but cannot conclude any relationship between dreads and granules.

If it is indeed the case that OYT1 and R-1 are the only dread-forming organisms, then we may identify genes potentially related to the production of dreads within the 373 genes (Supplementary Table S3) shared between the OYT1 and R-1 genomes, which were not detected in the other FeOB genomes; however, given the close relationship between OYT1 and R-1, that these are only two genomes, and the possibility that other FeOB make dread-like structures, there is a considerable amount of uncertainty here. Within these 373 genes, we found CDS13–16 related to *bcsABCZ* genes, which are involved in extracellular cellulose production (Römling, 2002; Mazur and Zimmer, 2011). Genes involved in synthesis of UDP-activated glucose and in gluconeogenesis, which are necessary to cellulose synthesis, were also found in the OYT1 and R-1 genomes. The set of *bcsABCZ-*like genes was not found in the other FeOB genomes, suggesting that the *bcsABCZ*-like genes found in only dread-forming FeOB are involved in the production of dreads. As described above, the other *bcsB*-like gene (CDS9) was found with *xag*-like genes (CDS10–12) in the OYT1 and R-1 genomes. Further organic chemical analysis are needed to determine if dreads and stalks contain cellulose.

#### Role of Stalk and Dreads

Why would neutrophilic microaerophilic FeOB produce two different biomineral morphologies? Stalk production was previously proposed as a mechanism(s) for avoiding encrustation by iron oxides (Chan et al., 2009, 2011). The *Ferriphaselus* OYT1 and R-1 dread structures appear to be easily shed, and therefore could play a role for avoiding Fe encrustation, leaving the stalks to play other roles. Stalks are associated with iron microbial mat formation. In this context, the stalks can serve as an anchor to attach on solid surfaces, preventing cells from being washed away in streams, groundwater seeps, or deep-sea vents. Attachment can also keep cells in the preferred microenvironment within opposing concentration gradients of oxygen and iron. Indeed, in gradient cultures, stalk-forming FeOB can attach to a glass surface and make colonies consisting of stalks and cells (Kucera and Wolfe, 1957; Kato et al., 2014). Previous studies showed that anchored *Mariprofundus* PV-1 cells can move by lengthening the stalks (Chan et al., 2011; Krepski et al., 2013). Such lifestyle of attachment and movement using extracellular slime from one side of a cell have been also reported from myxobacteria (Wolgemuth et al., 2002) and cyanobacteria (Hoiczyk and Baumeister, 1998).

Dreads may be more akin to the granular iron oxides produced by the many non-stalk-forming iron-oxidizers (e.g., Emerson and Moyer, 1997; Miot et al., 2009a,b). In contrast to the larger stalks, dreads and granules are small (∼10 s nm) and therefore likely mobile. So, while stalks represent a sink for Fe and any adsorbed or coprecipitated elements, the nanoparticulate dreads and granules (i.e., nanoBIOS) can be carried by groundwater and surface water, thereby redistributing Fe, along with associated heavy metals (e.g., Cu, Zn) and nutrients (e.g., C and P). In the environment, granular nanoBIOS are difficult to distinguish from abiotic oxides; however, the tapered, segmented dread morphology is much more recognizable and could therefore be used to help trace the fate of biogenic Fe oxides.

#### Attachment, Motility and Chemotaxis

Pili are involved in attachment on solid surfaces, twitching motility, and then in colonization or biofilm formation (Mattick, 2002). Genes related to Type IV pilus system proteins (PilACGHIJMNOPQSR) were found in the genomes of OYT1 and R-1, which are both organisms that are able to colonize the glass wall surface of a culture tube. Although direct evidence of the presence of pili of OYT1 and R-1 is still lacking, they have the genetic potential to produce pili, which could play a role in the attachment to surfaces.

The flagellum is one of the common constructs for bacterial mobility (Jarrell and McBride, 2008), and also plays a role in chemotaxis (Porter et al., 2011) and biofilm formation (Belas, 2014). Genes for all core proteins (Fli, Flg, and Flh proteins) of the bacterial flagellum complex (Macnab, 2003) were found in the OYT1 genome, and those except FliC were found in the R-1 genome. The presence of a polar flagellum has been microscopically confirmed in OYT1 (Kato et al., 2014), but not R-1. Genes for chemotaxis proteins (CheABDRWVYZ) and chemoreceptor proteins including aerotaxis receptor (Aer) were also found in the OYT1 and R-1 genomes. The genetic potential of aerotaxis is consistent with the obligate microaerophilic lifestyle of OYT1 and R1.

### Genetic Marker Candidates for Neutrophilic Microaerophilic Iron-oxidizing Bacteria

To date, the presence and abundance of neutrophilic microaerophilic FeOB in a given environment has been based mainly on the detection of 16S rRNA genes closely related to the cultured FeOB (Kato et al., 2009a,b; Wang et al., 2009; Fleming et al., 2013). However, 16S rRNA genes do not necessarily correlate to metabolism. Although common microaerophilic FeOB fall within phylogenetic clusters (Gallionellales, Zetaproteobacteria), there is some uncertainty as to whether these clusters contain some non-FeOB. Gallionellales appears to be mostly comprised of FeOB, including many isolates of freshwater FeOB; however, the order also contains the nitrite-oxidizing bacterium ‘*Candidatus Nitrotoga arctica’* (Alawi et al., 2007), though culture-independent work suggests that Nitrotoga relatives form a distinguishable subclade (Lücker et al., 2015). In addition, there may be FeOB that we do not recognize by 16S rRNA gene identity because we have no related culture. If reliable functional gene-based markers could be developed for FeOB, we would be able to more confidently assess the spatial distribution, abundance, and activity of the FeOB in the environment. Below, we evaluate candidates of genetic markers for neutrophilic microaerophilic FeOB.

The ideal Fe oxidation genetic marker would be (1) required for Fe oxidation (ideally the Fe oxidase), (2) present in all neutrophilic microaerophilic FeOB, and (3) fall into a monophyletic cluster that does not include other organisms (non-FeOB). We found 15 genes (CDS2–12 and 17–20) that meet criteria 2 and 3. We do not discuss CDS17–20 in detail, though the clustering (Supplementary Figure S9) suggests they are related to some unique physiological characteristics of the FeOB. Here we focus on the *act* genes (CDS2–8) and *cyc2* (CDS1), as promising candidates for genetic markers; as electron transport genes, they may meet the first criterion. Cyc2 is the leading candidate for an Fe oxidase; all neutrophilic microaerophilic FeOB have the gene, and the function has been demonstrated in an acidophilic FeOB. While the microaerophilic Cyc2 do form a phylogenetic cluster (**Figure 3**), it is not monophyletic, i.e., the cluster contains organisms with other metabolisms, though it is certainly possible, just not proven that these other organisms oxidize Fe(II). In contrast, the *act* genes are monophyletic (**Figure 4**, Supplementary Figure S1). They are present in all neutrophilic microaerophilic FeOB with genomes available, but the role in Fe oxidation is not clear. If it can be shown that Cyc2 and ACIII are necessary for Fe oxidation, then these genes could serve as genetic markers of microaerophilic Fe oxidation.

In summary, we have analyzed newly sequenced genomes from freshwater stalk-formers *Ferriphaselus* sp. OYT1 and R-1 to understand how microaerophilic FeOB function and contribute to biogeochemical cycling. Comparisons with the genomes of five existing Betaproteobacterial and Zetaproteobacterial FeOB show us patterns of gene presence and absence that advance our working hypotheses on Fe oxidation and biomineralization mechanisms. Analyzing genes specific to the genomes of the stalk-formers *Ferriphaselus* sp. and *Mariprofundus* sp. gives us some first insights into genes potentially involved in stalk structure, which would be useful in tracking Fe microbial mat formation. Differences between FeOB genomes, e.g., C, N, and S cycling genes and terminal oxidases, suggest that different FeOB are adapted to particular niches and contribute to other biogeochemical cycles in varied ways. Although we still need to demonstrate the function of the genes highlighted here, these genomic analyses begin to focus our picture of FeOB roles and mechanisms in Fe cycling in both freshwater and marine environments.

### Description of *Ferriphaselus globulitus* sp. nov.

*Ferriphaselus globulitus* [glo.bu.li’tus. L. dim. n. globulus, a small sphere, globule; L. suff. -atus, suffix used with the sense provided with; N.L. masc. adj. globulitus, having small globules].

Cells are curved rods, or bean-shaped, 1.8–2.1 μm in length. Motile. Gram-negative. Do not form spores. Mesophilic and neutrophilic. Microaerobic, growing with opposing gradients of Fe(II) and O_2_. Autotrophic. Capable of oxidizing Fe(II) as an energy source. Do not utilize thiosulfate, sulfide, nitrite, Mn(II), pyruvate, glucose or acetate as an energy source. Produces extracellular dreadlock-like iron oxides, in addition to twisted stalks. The major cellular fatty acids are C18:0, C16:0, C16:1v7c and/or C16:1v6c. Grows at 10–35°C (optimally at 25–30°C) and pH 5.6–7.0 (optimally at pH 5.6–6.1). Grows at low salt concentrations, below 0.3 g NaCl per L. The doubling time is 15 h. The type strain is R-1, isolated from an iron-rich floc in a groundwater seep in Christiana Creek, Newark, Delaware, United States of America. The total DNA G + C content of the type strain is 62.4 mol%.

## Author Contributions

SK and CC designed the study, analyzed the data, and wrote the paper. SK performed most of the experimentation, unless otherwise noted. DP performed the SEM imaging. STK cultured R-1 for various analyses. KO, MH, NS, and TW contributed to genome sequencing and annotation.

## Conflict of Interest Statement

The authors declare that the research was conducted in the absence of any commercial or financial relationships that could be construed as a potential conflict of interest.
