# Supplementary material for: Comparative Genomic Insights into Ecophysiology of Neutrophilic, Microaerophilic Iron Oxidizing Bacteria
Source: Front Microbiol. 2015 Nov 13;6:1265. doi: 10.3389/fmicb.2015.01265 (PMC4643136; doi:10.3389/fmicb.2015.01265)
Supplement: Supplementary file 2 [file Data_Sheet_1.PDF]

Supplementary Material

Comparative genomic insights into ecophysiology of neutrophilic, microaerophilic iron oxidizing bacteria

Shingo Kato*, Moriya Ohkuma, Deborah H. Powell, Sean T. Krepski, Kenshiro Oshima, Masahira Hattori, Nicole Shapiro, Tanja Woyke, Clara S. Chan*

*** Correspondence:** Shingo Kato: skato@jcm.riken.jp; Clara Chan: cschan@udel.edu

Supplementary Figure legends

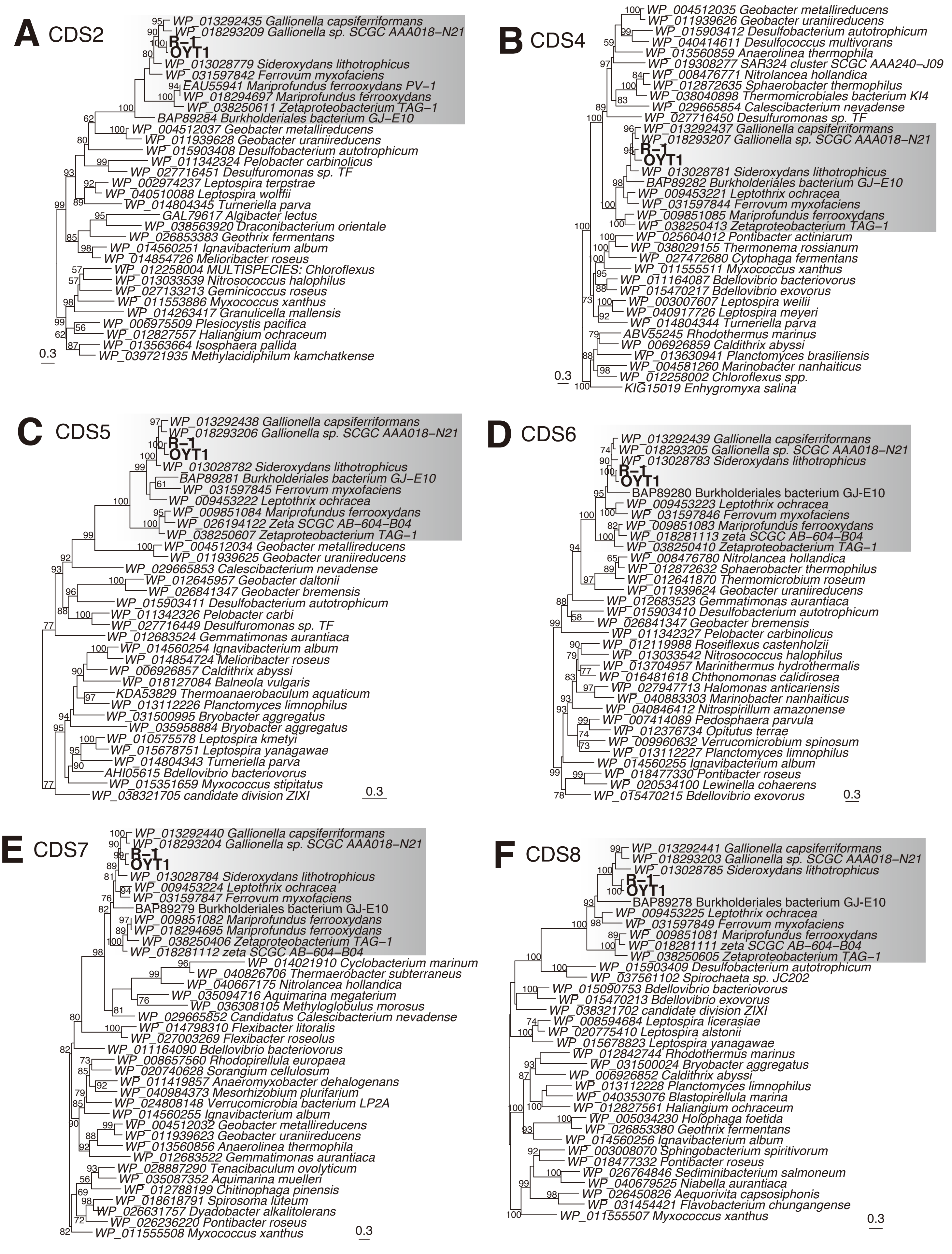

Figure S1. Phylogenetic trees of proteins related to (A) CDS2 (ActA-like), (B) CDS4 (ActB2-like), (C) CDS5 (ActC-like), (D) CDS6 (ActD-like), (E) CDS7(ActE-like), and (F) CDS8 (ActF-like). Bootstrap values (>50 of 100 replicates) are shown at the branch points. Each of gray boxes indicates a clade including FeOB cultivates and SAGs of *Betaproteobacteria* and *Zetaproteobacteria*.

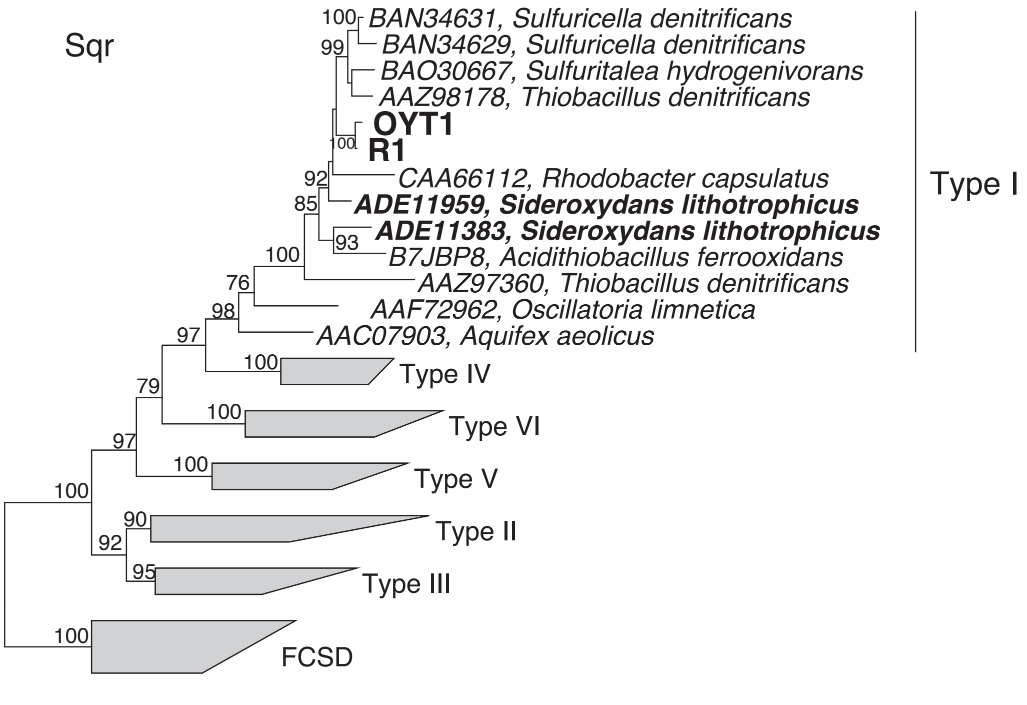

Figure S2. Phylogenetic tree of Sqr. Bootstrap values (>50 of 100 replicates) are shown at the branch points. FCSD, flavocytochrome *c*:sulfide dehydrogenase.

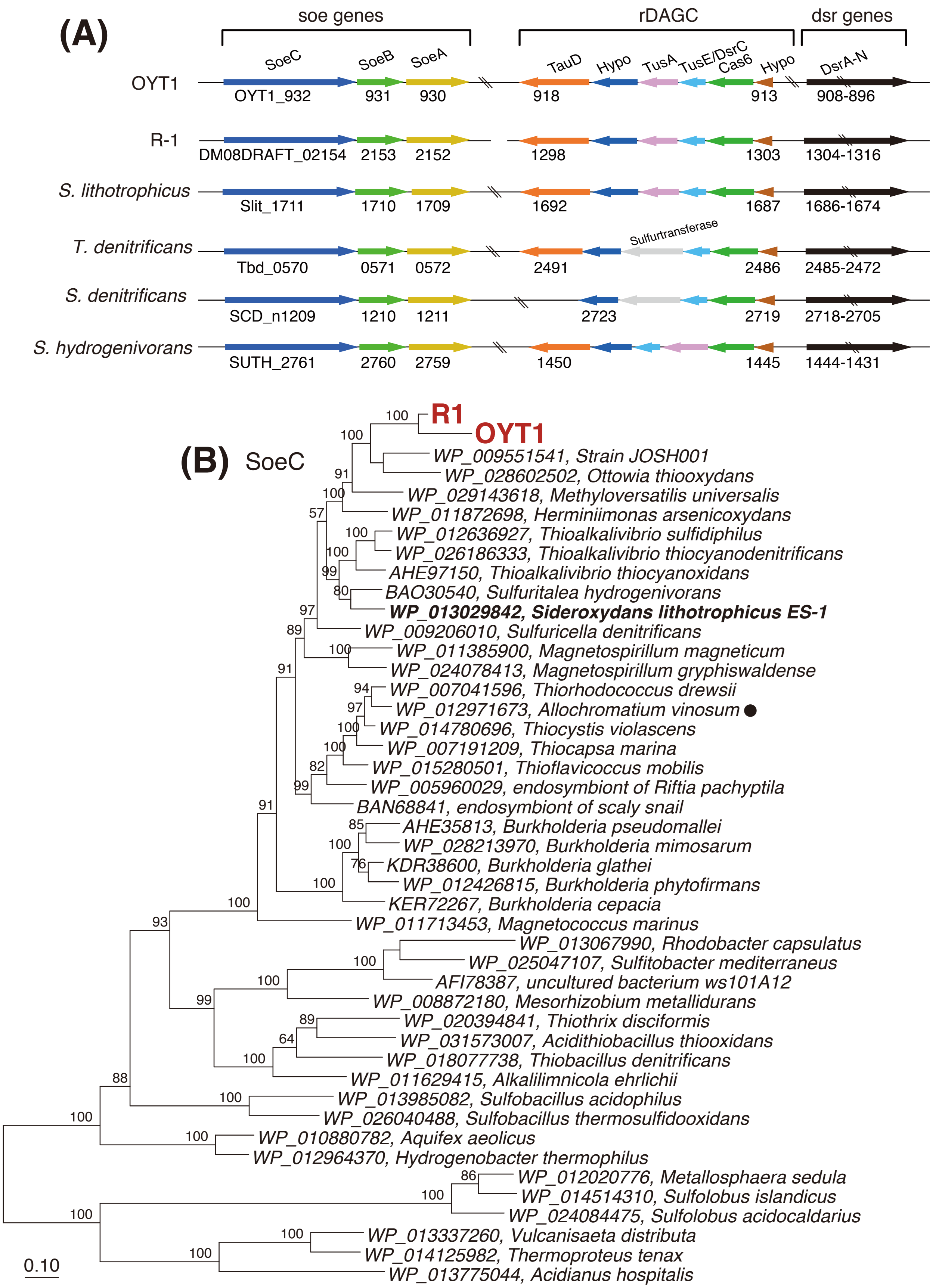

Figure S3. CDS related to sulfur oxidation. (A) Gene orders of Dsr and Soe cluster, and rDAGC. Homologs among the genomes are shown in the same color. (B) Phylogenetic tree of SoeC. Bootstrap values (>50 of 100 replicates) are shown at the branch points. Filled circle following the bacterial name indicate that biochemical function of the enzyme have been characterized.

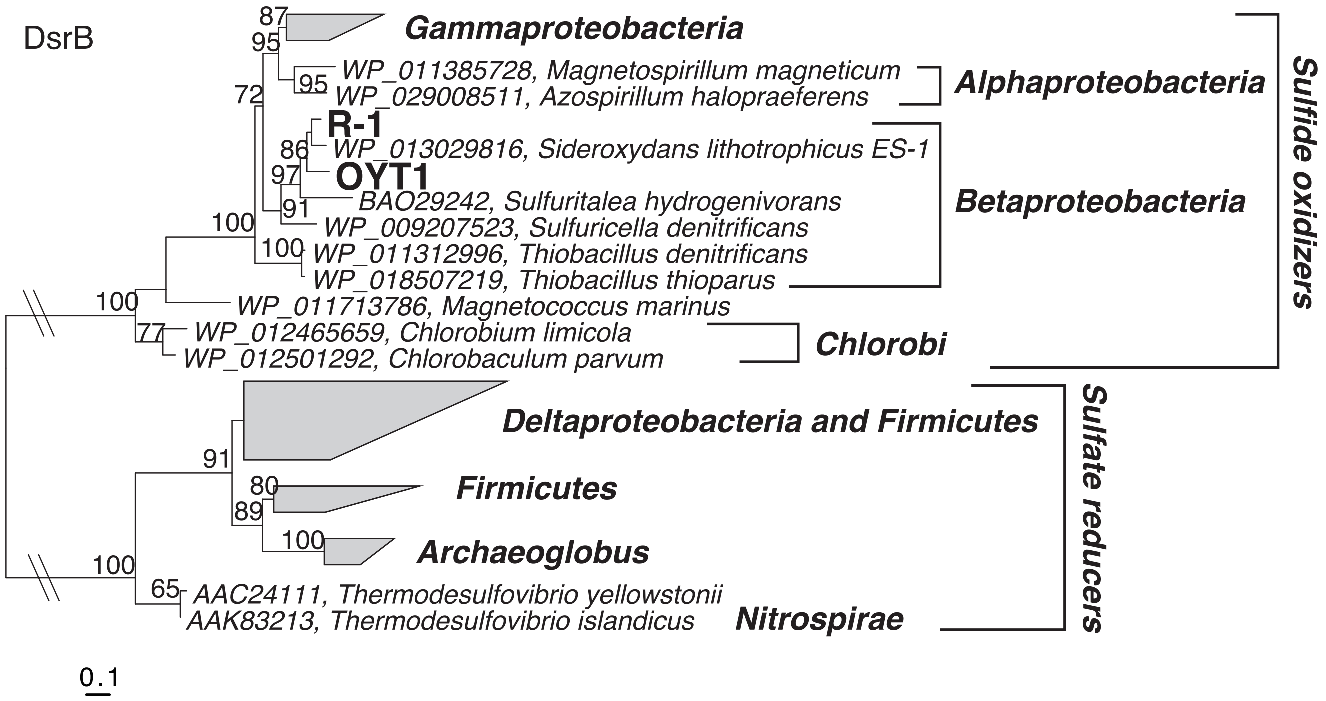

Figure S4. Phylogenetic tree of DsrB. Bootstrap values (>50 of 100 replicates) are shown at the branch points.

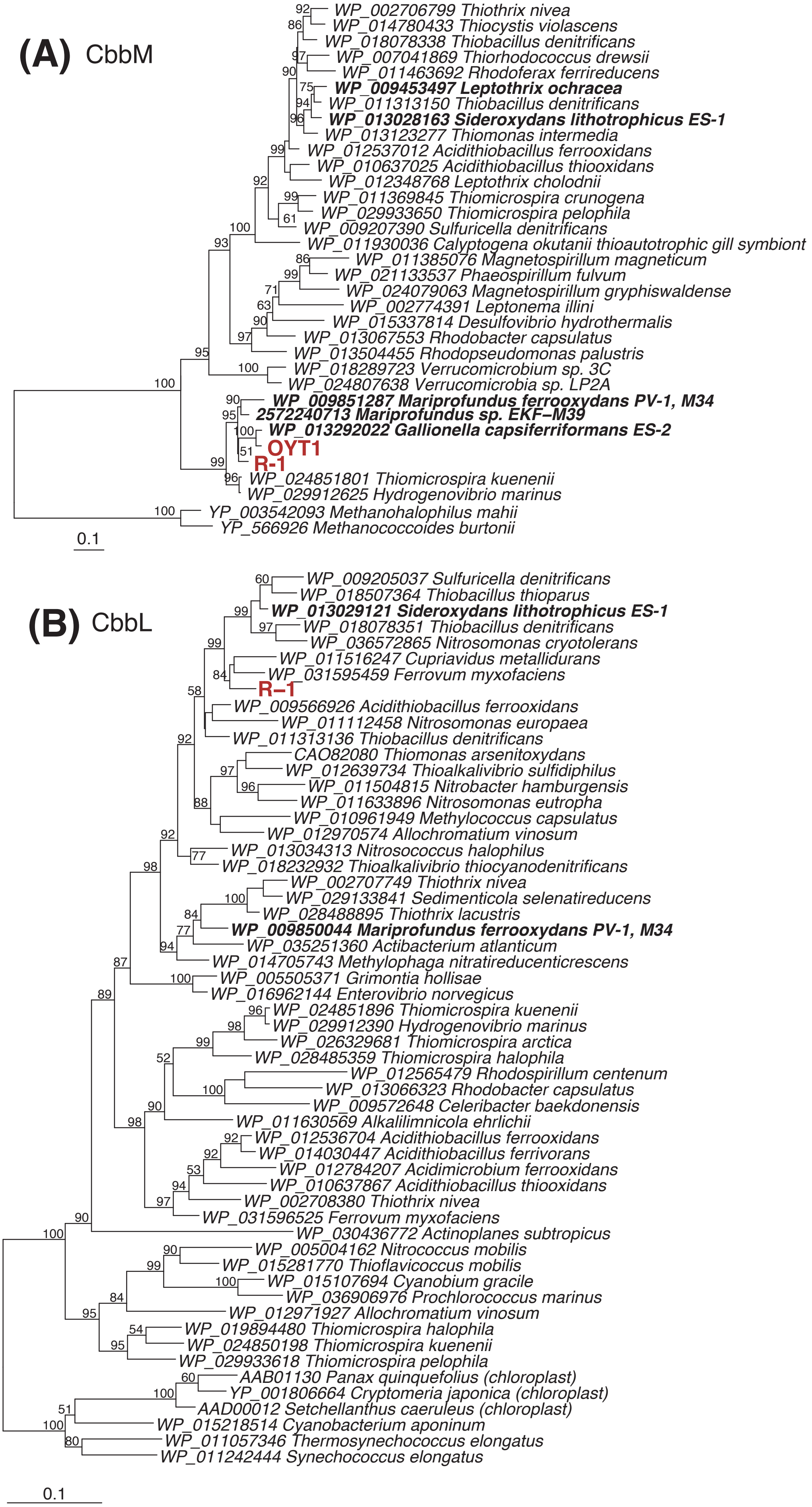

Figure S5. Phylogenetic tree of (A) CbbM and (B) CbbL. Bootstrap values (>50 of 100 replicates) are shown at the branch points. Betaproteobacterial and zetaproteobacterial neutrophilic FeOB are shown in bold.

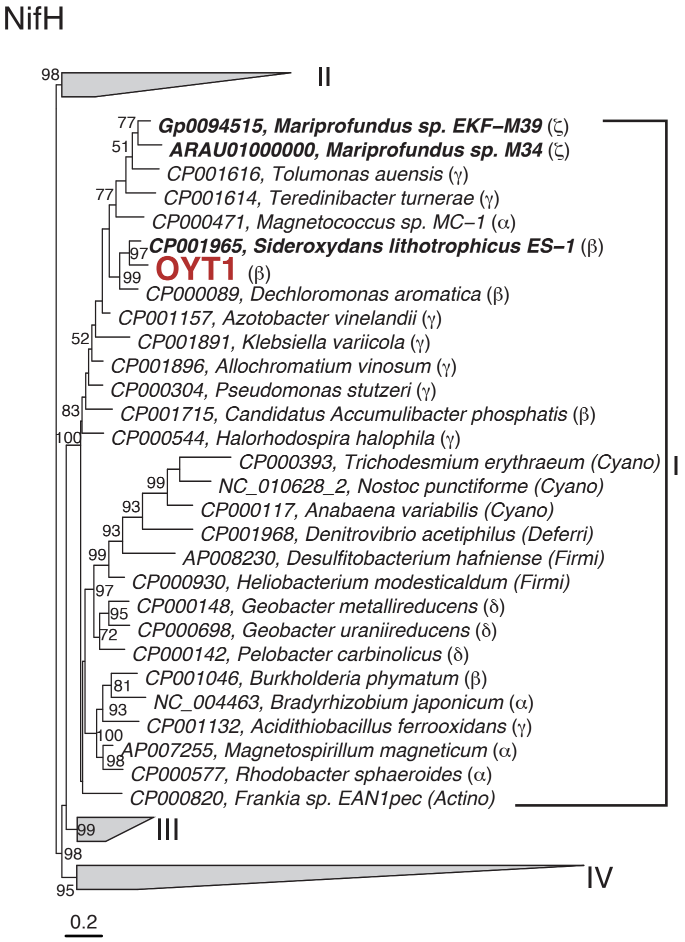

Figure S6. Phylogenetic tree of NifH. Bootstrap values (>50 of 100 replicates) are shown at the branch points. FeOB are shown in bold. Letters in the parentheses following the bacterial names indicate their phyla (Cyano, *Cyanobacteria*; Deferri, *Deferribacteres*; Firmi, *Firmicutes*; Actino, *Actinobacteia*) or proteobacterial classes.

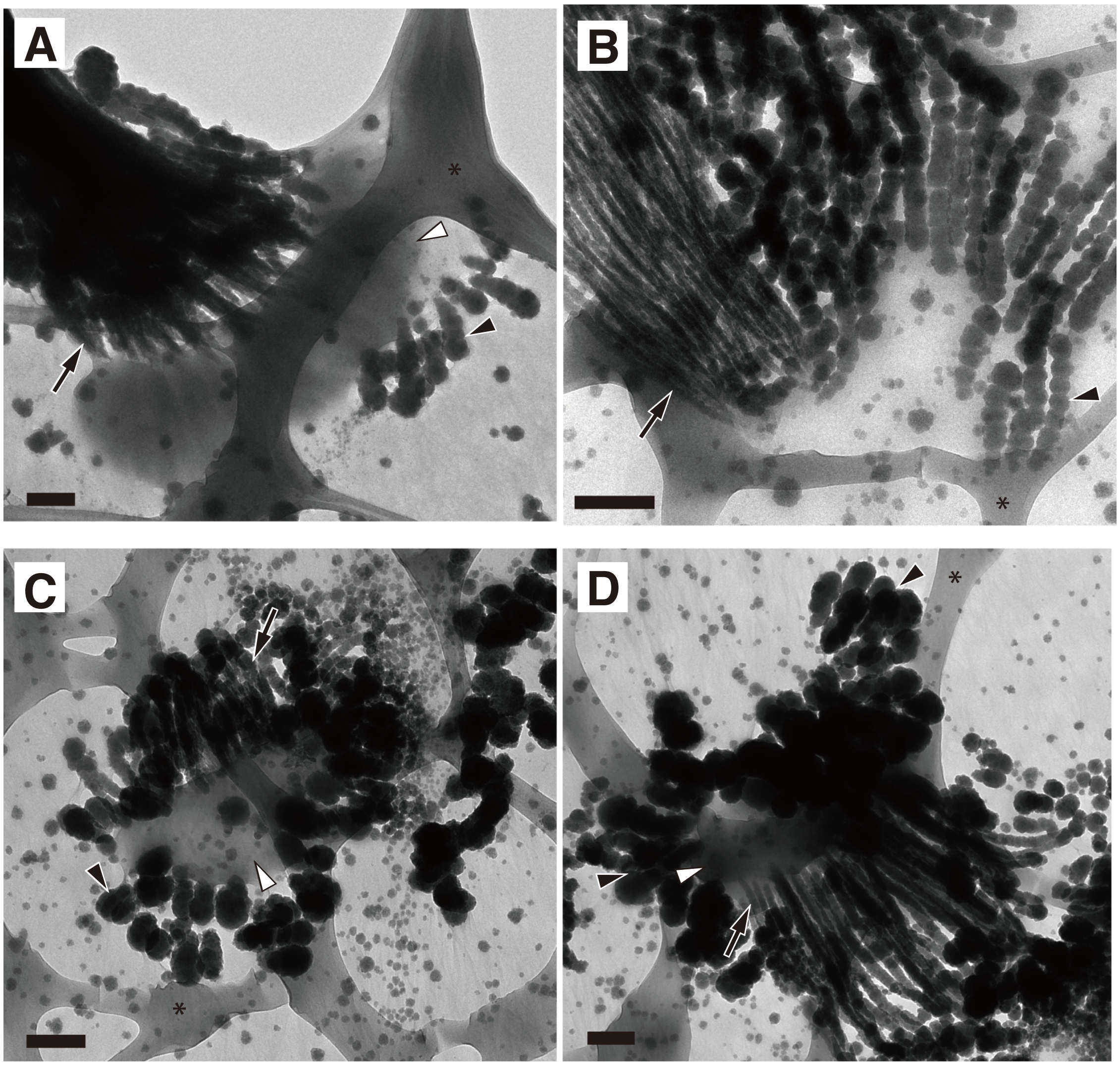

Figure S7. Transmission electron micrographs of strains (A and B) OYT1 and (C and D) R-1. Asterisk (*) indicates lacey carbon on the TEM grids. White arrowheads, cells; black arrowheads, dreads; black arrows, stalks. Bars, 200 nm.

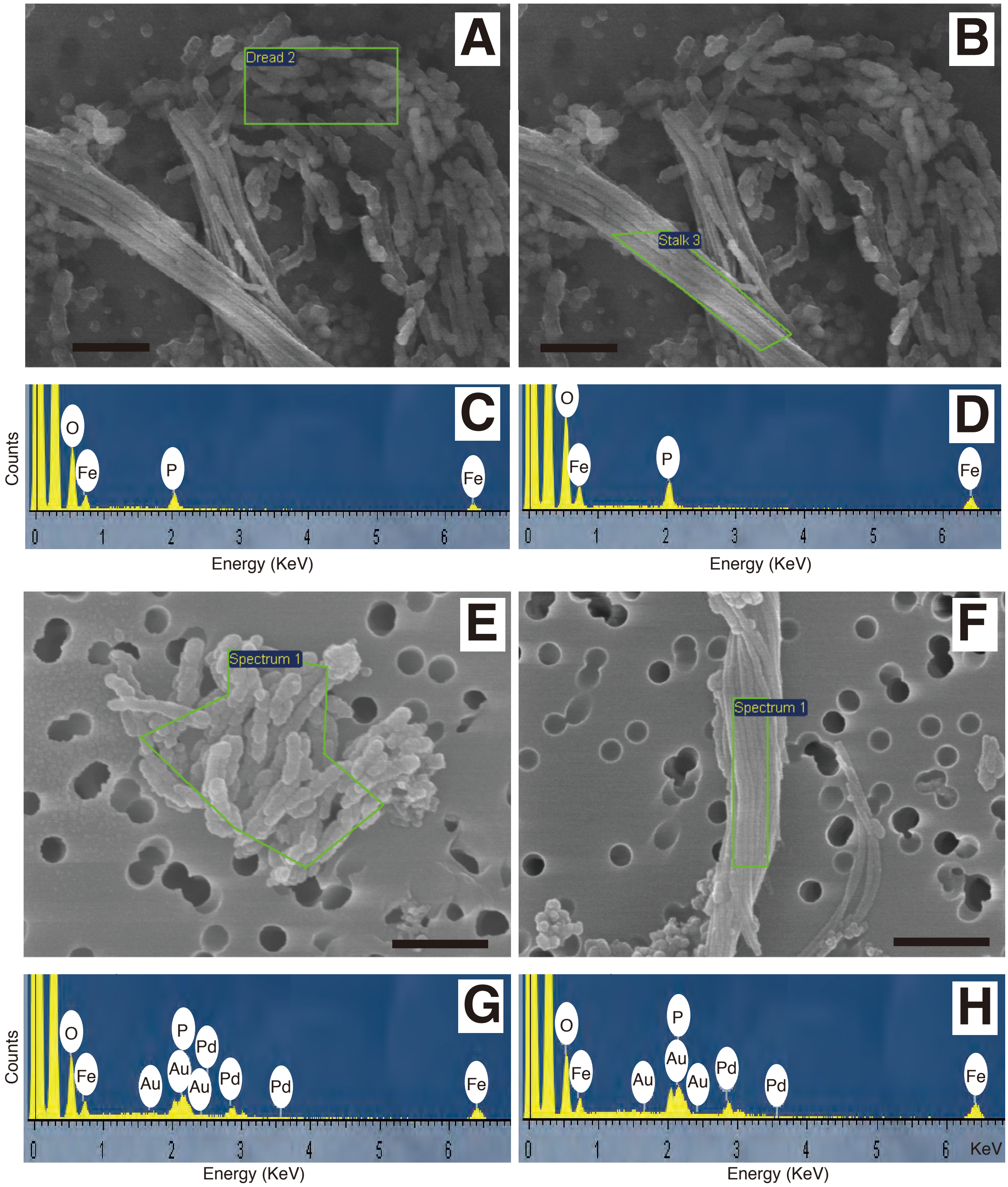

Figure S8. SEM-EDX analysis of dreads and stalks. The results of (A-D) OYT1 with carbon coating and (E-H) R-1 with gold/palladium. SEM images of (A and E) dreads and (B and F) stalks. Green box indicate the analyzed areas. Scale bars, 1 µm. EDX results of (C and G) dreads and (D and H) stalks. (G and H) Au/Pd are originated from the coating materials.

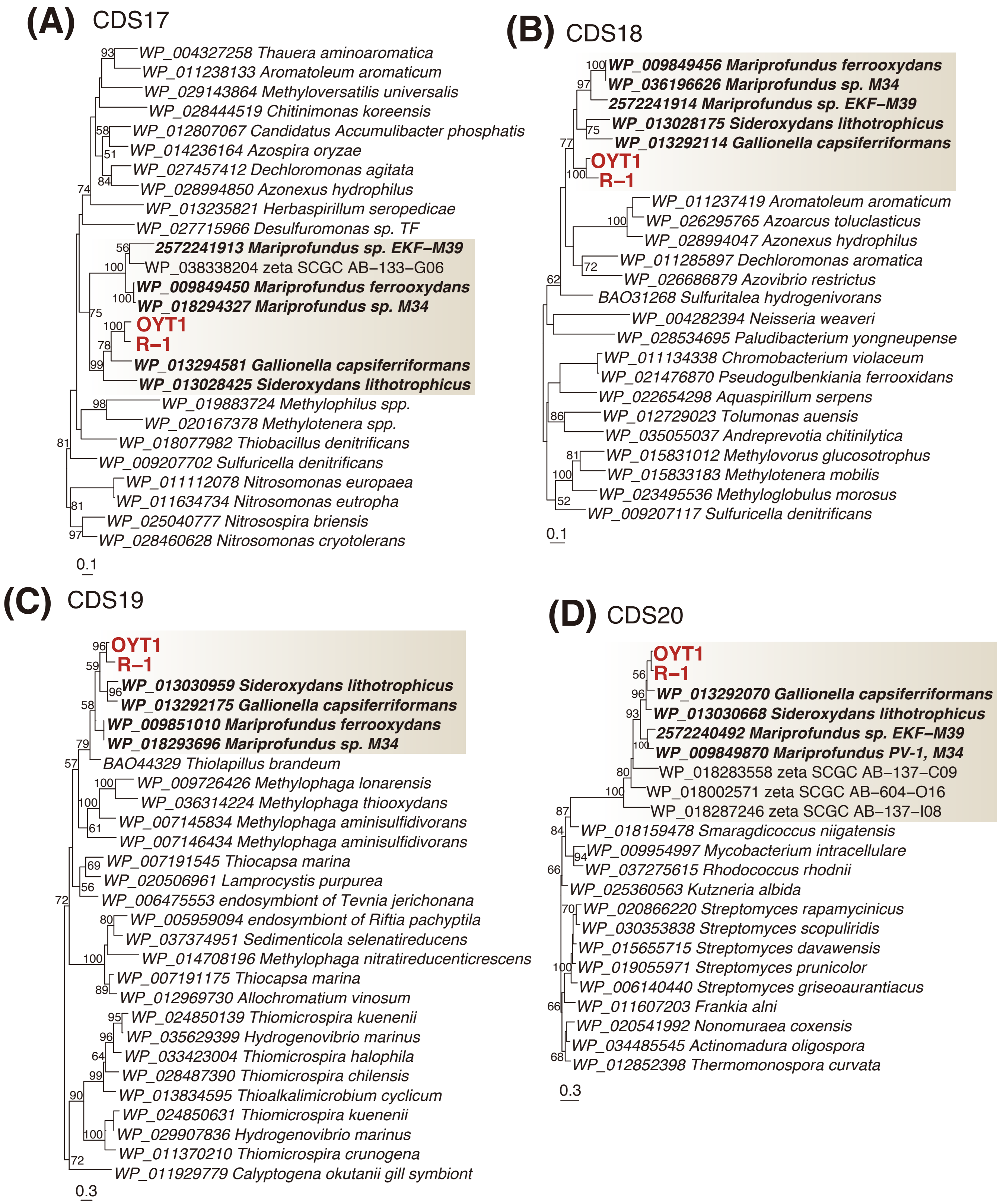

Figure S9. Phylogenetic tree for proteins related to (A) CDS17, (B) CDS18, (C) CDS19 and (D) CDS20. Bootstrap values (>50 of 100 replicates) are shown at the branch points. Betaproteobacterial and zetaproteobacterial neutrophilic FeOB are shown in bold. The gray box indicates a clade including FeOB isolates of *Gallionellales* and *Zetaproteobacteria* and zetaproteobacterial SAGs.

Supplementary Video legends

Video. S1. Series of microscopic images of stalks and dreads formation by OYT1 in a microslide growth chamber. Total time, 3 h; time interval, 5 min; image width, 222 μm. The image capturing was started 1 h after each inoculation.

Supplementary Table legends

Table S1. FAME analysis of fatty acids from OYT1 and R-1 cells.

	R-1	OYT1
10:0 3OH	0.78	3.35
unknown 11.825	0.84	-
12:0	4.57	2.18
14:0	-	1.59
15:0	-	1.22
15:1 w5c	1.84	-
16:0 N alcohol	7.41	0.89
Sum in feature 3[1]	9.34	59.93
16:1 w5c	-	1.18
16:0	13.94	22.23
17:0	0.67	-
17:1 w6c	-	0.68
18:3 w6c (6,9,12)	0.72	0.8
18:1 w9c	-	1.42
18:1 w7c	0.55	2.24
18:0	59.35	2.28

[1] 16:1w7c/16:1 w6c

R-1, this study; OYT1 from Kato et al., 2014

Table S2. List of CDS that zetaproteobacterial genes appear in the top 10 blast hits. Symbols: +, found in the top 10 hits; -, not found in the top 10 hits.

OYT1 tag#	R-1 tag#	Annotation*	Sideroxydans ES-1	Gallionella ES-2	Mariprofundus PV-1, M34, or EKF-M39	Note
OYT1_00079	DM08DRAFT_02265	hypothetical protein	+	+	+	CDS8 (actF-like)
OYT1_00080	DM08DRAFT_02264	Cytochrome c_6	+	+	+	CDS7 (actE-like)
OYT1_00081	DM08DRAFT_02263	hypothetical protein	+	+	+	CDS6 (actD-like)
OYT1_00082	DM08DRAFT_02262	putative hydrogenase 2 *b* cytochrome subunit	+	+	+	CDS5 (actC-like)
OYT1_00083	DM08DRAFT_02261	Tetrathionate reductase subunit B precursor	+	+	+	CDS4 (actB2-like)
OYT1_00084	DM08DRAFT_02260	Polysulfide reductase chain A precursor	+	+	+	CDS3 (actB1-like)
OYT1_00085	DM08DRAFT_02259	Class III cytochrome *c* family protein	+	+	+	CDS2 (actA-like)
OYT1_00147	-	hypothetical protein	-	-	+	
OYT1_00164	DM08DRAFT_00350	hypothetical protein	-	-	+	CDS12 (xagD-like)
OYT1_00165	DM08DRAFT_00349	hypothetical protein	-	-	+	CDS11 (xagC-like)
OYT1_00166	DM08DRAFT_00348	Beta-monoglucosyldiacylglycerol synthase	-	-	+	CDS10 (xagB-like)
OYT1_00167	DM08DRAFT_00347	cellulose synthase regulator protein	-	-	+	CDS9 (bcsB-like)
OYT1_00168	DM08DRAFT_00346	hypothetical protein	+	+	+	CDS1 (cyc2-like)
OYT1_00424	DM08DRAFT_00510	FixH	+	+	+	
OYT1_00447	-	Response regulator SaeR	-	-	+	
OYT1_00490	DM08DRAFT_00581	Cytochrome c_1 precursor	+	-	+	
OYT1_00547	DM08DRAFT_00807	bifunctional RNase H/acid phosphatase	-	-	+	
OYT1_00611	DM08DRAFT_00644	Ribulose bisphosphate carboxylase	-	+	+	
OYT1_00612	DM08DRAFT_00645	Denitrification regulatory protein NirQ	+	+	+	
OYT1_00613	DM08DRAFT_00646	von Willebrand factor type A domain protein	+	+	+	
OYT1_00623	DM08DRAFT_00652	Lipopolysaccharide core biosynthesis protein RfaG	+	+	+	
OYT1_00629	-	L-glutamine:2-deoxy-scyllo-inosose aminotransferase	-	-	+	
OYT1_00630	DM08DRAFT_00132	Putative acetyltransferase EpsM	-	-	+	
OYT1_00632	-	N,N'-diacetylbacillosaminyl-diphospho-undecaprenol alpha-1,3-N-acetylgalactosaminyltransferase	-	-	+	
OYT1_00633	-	Putative glycosyltransferase EpsE	-	-	+	
OYT1_00634	-	Glycosyl transferase family 11	+	-	+	
OYT1_00635	-	hypothetical protein	-	-	+	
OYT1_00636	-	Demethylrebeccamycin-D-glucose O-methyltransferase	-	-	+	
OYT1_00639	-	Polysaccharide biosynthesis protein	-	-	+	
OYT1_00973	DM08DRAFT_01097	Inner spore coat protein H	-	-	+	
OYT1_00981	-	WLM domain protein	+	-	+	
OYT1_01041	-	Glucose-6-phosphate isomerase	-	+	+	
OYT1_01061	DM08DRAFT_01241	TLC ATP/ADP transporter	-	-	+	
OYT1_01141	DM08DRAFT_00299	hypothetical protein	+	-	+	
OYT1_01169	DM08DRAFT_00114	Outer membrane protein OprM precursor	+	+	+	
OYT1_01170	DM08DRAFT_00113	Macrolide export protein MacA	+	+	+	
OYT1_01171	DM08DRAFT_00112	Macrolide export ATP-binding/permease protein MacB	+	-	+	
OYT1_01172	DM08DRAFT_00111	Macrolide export ATP-binding/permease protein MacB	+	-	+	
OYT1_01292	-	hypothetical protein	-	-	+	
OYT1_01293	-	hypothetical protein	-	-	+	
OYT1_01294	-	hypothetical protein	-	-	+	
OYT1_01303	-	Putative phospholipase A1 precursor	-	+	+	
OYT1_01454	DM08DRAFT_00108	Membrane-bound lytic murein transglycosylase A precursor	+	+	+	CDS17
OYT1_01521	-	Bacteriohemerythrin	-	-	+	
OYT1_01534	DM08DRAFT_01856	muropeptide transporter	+	+	+	CDS18
OYT1_01537	DM08DRAFT_01848	Putative glucose-6-phosphate 1-epimerase	+	+	+	
OYT1_01541	DM08DRAFT_01844	Long-chain-fatty-acid--CoA ligase FadD15	-	-	+	
OYT1_01542	DM08DRAFT_01843	hypothetical protein	-	-	+	
OYT1_01543	DM08DRAFT_01842	Fatty acyl-CoA reductase	-	-	+	
OYT1_01557	DM08DRAFT_01683	hypothetical protein	+	-	+	
OYT1_01613	-	hypothetical protein	-	+	+	
OYT1_01652	DM08DRAFT_01365	L-glyceraldehyde 3-phosphate reductase	+	+	+	
OYT1_01824	DM08DRAFT_01098	hypothetical protein	-	-	+	
OYT1_01825	DM08DRAFT_01097	Inner spore coat protein H	-	-	+	
OYT1_01844	-	Ferredoxin-1	+	-	+	
OYT1_01846	-	hypothetical protein	+	-	+	
OYT1_01852	-	proteasome-activating nucleotidase	-	-	+	
OYT1_01962	DM08DRAFT_00885	hypothetical protein	-	+	+	
OYT1_02027	DM08DRAFT_01628	hypothetical protein	-	-	+	
OYT1_02038	DM08DRAFT_00156	Ubp3 associated protein Bre5	+	+	+	
OYT1_02152	-	hypothetical protein	-	-	+	
OYT1_02155	DM08DRAFT_00721	hypothetical protein	-	-	+	
OYT1_02332	DM08DRAFT_00208	Cyclic di-GMP phosphodiesterase Gmr	+	-	+	
OYT1_02351	DM08DRAFT_02295	hypothetical protein	+	+	+	CDS19
OYT1_02368	-	Na(+)/H(+) antiporter NhaG	+	-	+	
OYT1_02422	-	Cyclic di-GMP phosphodiesterase response regulator RpfG	-	-	+	
OYT1_02424	DM08DRAFT_00646	Aerobic respiration control sensor protein ArcB	-	-	+	
OYT1_02489	DM08DRAFT_01432	hypothetical protein	-	-	+	
OYT1_02525	DM08DRAFT_00129	NifZ domain protein	+	-	+	
OYT1_02529	DM08DRAFT_00133	2-isopropylmalate synthase	+	-	+	
OYT1_02533	DM08DRAFT_00885	hypothetical protein	+	-	+	
OYT1_02581	-	Outer membrane protein A precursor	-	-	+	
OYT1_02608	DM08DRAFT_00151	Nitrogenase molybdenum-iron protein beta chain	+	-	+	
OYT1_02621	DM08DRAFT_02432	Soluble pyridine nucleotide transhydrogenase	+	+	+	CDS20
OYT1_02661	-	hypothetical protein	-	-	+	
OYT1_02662	-	Mor transcription activator family protein	+	-	+	
OYT1_02663	-	hypothetical protein	+	-	+	
-	DM08DRAFT_01435	Tyrosine-protein phosphatase YwqE	-	-	+	
-	DM08DRAFT_01437	hypothetical protein	-	-	+	
-	DM08DRAFT_01647	Phage-related minor tail protein	+	-	+	
-	DM08DRAFT_01655	Mu-like prophage I protein	-	-	+	
-	DM08DRAFT_02312	hypothetical protein	-	+	+	

* Annotation of OYT1 CDS by Prokka; lines coded in color are focused on the main text

Table S3. List of CDS shared between the OYT1 and R-1 genomes, but not found in the other neutrophilic microaerophilic FeOB genomes.

OYT1 tag#	R-1 tag#	Annotation*	Note
OYT1_00010	DM08DRAFT_01147	Signal transduction histidine-protein kinase BarA	
OYT1_00012	DM08DRAFT_02371	Thermonuclease precursor	
OYT1_00026	DM08DRAFT_02041	Proline/betaine transporter	
OYT1_00031	DM08DRAFT_01260	putative adenylyltransferase/sulfurtransferase MoeZ	
OYT1_00033	DM08DRAFT_01262	Hydroxyacylglutathione hydrolase	
OYT1_00040	DM08DRAFT_02281	hypothetical protein	
OYT1_00042	DM08DRAFT_02279	hypothetical protein	
OYT1_00043	DM08DRAFT_02278	Bacteriohemerythrin	
OYT1_00044	DM08DRAFT_02277	cofactor-independent phosphoglycerate mutase	
OYT1_00067	DM08DRAFT_00723	dTDP-3-amino-3,6-dideoxy-alpha-D-galactopyranose 3-N-acetyltransferase	
OYT1_00070	DM08DRAFT_01428	UDP-glucose 4-epimerase	
OYT1_00075	DM08DRAFT_02269	Glutamine amidotransferases class-II	
OYT1_00103	DM08DRAFT_02241	Transcriptional regulatory protein AfsQ1	
OYT1_00104	DM08DRAFT_02240	Putative anti-sigma factor antagonist	
OYT1_00128	DM08DRAFT_01822	hypothetical protein	
OYT1_00147	DM08DRAFT_00370	hypothetical protein	
OYT1_00148	DM08DRAFT_00369	Roadblock/LC7 domain protein	
OYT1_00149	DM08DRAFT_00368	hypothetical protein	
OYT1_00150	DM08DRAFT_00367	hypothetical protein	
OYT1_00152	DM08DRAFT_00365	hypothetical protein	
OYT1_00164	DM08DRAFT_00350	hypothetical protein	
OYT1_00165	DM08DRAFT_00349	hypothetical protein	
OYT1_00167	DM08DRAFT_00347	cellulose synthase regulator protein	
OYT1_00168	DM08DRAFT_00346	hypothetical protein	
OYT1_00173	DM08DRAFT_00341	Lipopolysaccharide-assembly, LptC-related	
OYT1_00174	DM08DRAFT_00340	Lipopolysaccharide export system protein LptA precursor	
OYT1_00187	DM08DRAFT_00328	hypothetical protein	
OYT1_00188	DM08DRAFT_00327	Photosynthetic apparatus regulatory protein RegA	
OYT1_00190	DM08DRAFT_00326	Gram-negative bacterial tonB protein	
OYT1_00191	DM08DRAFT_00325	Biopolymer transport protein ExbB	
OYT1_00192	DM08DRAFT_00324	Biopolymer transport protein ExbD	
OYT1_00196	DM08DRAFT_00321	Multidrug resistance protein MdtA precursor	
OYT1_00198	DM08DRAFT_00319	hypothetical protein	
OYT1_00210	DM08DRAFT_00479	6-phosphogluconate dehydrogenase, NADP(+)-dependent, decarboxylating	
OYT1_00211	DM08DRAFT_00478	hypothetical protein	
OYT1_00215	DM08DRAFT_00472	Response regulator PleD	
OYT1_00216	DM08DRAFT_00471	hypothetical protein	
OYT1_00228	DM08DRAFT_00385	hypothetical protein	
OYT1_00230	DM08DRAFT_00383	47 kDa outer membrane protein precursor	
OYT1_00238	DM08DRAFT_00292	Flagellar assembly factor FliW	
OYT1_00289	DM08DRAFT_00254	STAS domain protein	
OYT1_00290	DM08DRAFT_00253	putative phospholipid-binding protein MlaC precursor	
OYT1_00294	DM08DRAFT_00249	Nitrogen assimilation regulatory protein	
OYT1_00295	DM08DRAFT_00248	Type II secretion system protein D precursor	
OYT1_00296	DM08DRAFT_00247	cellulose synthase subunit BcsC	bcsC-like
OYT1_00299	DM08DRAFT_00244	hypothetical protein	
OYT1_00300	DM08DRAFT_00243	hypothetical protein	
OYT1_00302	DM08DRAFT_00241	hypothetical protein	
OYT1_00303	DM08DRAFT_00240	hypothetical protein	
OYT1_00310	DM08DRAFT_00233	Phytochrome-like protein cph2	
OYT1_00315	DM08DRAFT_00228	O-Antigen ligase	
OYT1_00316	DM08DRAFT_00227	Type II secretion system protein G precursor	
OYT1_00317	DM08DRAFT_00226	hypothetical protein	
OYT1_00318	DM08DRAFT_00225	hypothetical protein	
OYT1_00319	DM08DRAFT_00224	hypothetical protein	
OYT1_00320	DM08DRAFT_00223	hypothetical protein	
OYT1_00321	DM08DRAFT_00222	Competence protein A	
OYT1_00322	DM08DRAFT_00221	Fimbrial assembly protein (PilN)	
OYT1_00323	DM08DRAFT_00220	hypothetical protein	
OYT1_00324	DM08DRAFT_00219	hypothetical protein	
OYT1_00325	DM08DRAFT_00218	Type II secretion system protein D precursor	
OYT1_00327	DM08DRAFT_00216	Tetratricopeptide repeat	
OYT1_00335	DM08DRAFT_01482	hypothetical protein	
OYT1_00341	DM08DRAFT_01862	Cyclic di-GMP phosphodiesterase Gmr	
OYT1_00348	DM08DRAFT_01869	hypothetical protein	
OYT1_00377	DM08DRAFT_01886	Signal transduction histidine-protein kinase/phosphatase DegS	
OYT1_00383	DM08DRAFT_01442	flagellar protein FlaG	
OYT1_00384	DM08DRAFT_01443	Flagellar hook-associated protein 2	
OYT1_00385	DM08DRAFT_01444	Flagellar protein FliS	
OYT1_00387	DM08DRAFT_01446	Flagellar hook-length control protein FliK	
OYT1_00389	DM08DRAFT_01448	Flagellar brake protein YcgR	
OYT1_00405	DM08DRAFT_01464	hypothetical protein	
OYT1_00408	DM08DRAFT_01467	hypothetical protein	
OYT1_00409	DM08DRAFT_01468	hypothetical protein	

OYT1_00413	DM08DRAFT_01472	Sensor histidine kinase LiaS	
OYT1_00438	DM08DRAFT_00616	hypothetical protein	
OYT1_00460	DM08DRAFT_00589	hypothetical protein	
OYT1_00461	DM08DRAFT_00588	AsmA family protein	
OYT1_00463	DM08DRAFT_01750	Phytochrome-like protein cph2	
OYT1_00476	DM08DRAFT_00530	Flagellar biosynthetic protein FliU	
OYT1_00479	DM08DRAFT_00528	Colicin I receptor precursor	
OYT1_00489	DM08DRAFT_00582	Cytochrome *b*	
OYT1_00490	DM08DRAFT_00581	Cytochrome c_1 precursor	
OYT1_00510	DM08DRAFT_00840	Cell division protein FtsL	
OYT1_00529	DM08DRAFT_00824	hypothetical protein	
OYT1_00536	DM08DRAFT_00818	hypothetical protein	
OYT1_00540	DM08DRAFT_00814	Putative uroporphyrinogen-III C-methyltransferase	
OYT1_00541	DM08DRAFT_00813	putative protoheme IX biogenesis protein	
OYT1_00545	DM08DRAFT_00809	Cyclic di-GMP phosphodiesterase Gmr	
OYT1_00547	DM08DRAFT_00807	bifunctional RNase H/acid phosphatase	
OYT1_00549	DM08DRAFT_00805	hypothetical protein	
OYT1_00558	DM08DRAFT_01523	hypothetical protein	
OYT1_00559	DM08DRAFT_01522	hypothetical protein	
OYT1_00566	DM08DRAFT_01161	putative transcriptional regulatory protein pdtaR	
OYT1_00567	DM08DRAFT_01162	Nitrate transport protein NrtA precursor	
OYT1_00568	DM08DRAFT_01163	Bicarbonate transport system permease protein CmpB	
OYT1_00569	DM08DRAFT_01164	Bicarbonate transport ATP-binding protein CmpD	
OYT1_00601	DM08DRAFT_00634	hypothetical protein	
OYT1_00602, OYT1_02510	DM08DRAFT_00635	Mannose-1-phosphate guanylyltransferase RfbM	
OYT1_00619	DM08DRAFT_00648	hypothetical protein	
OYT1_00620	DM08DRAFT_00649	hypothetical protein	
OYT1_00621	DM08DRAFT_00650	Cyclic di-GMP phosphodiesterase Gmr	
OYT1_00622	DM08DRAFT_00651	GtrA-like protein	
OYT1_00626	DM08DRAFT_00655	O-Antigen ligase	
OYT1_00646	DM08DRAFT_00681	hypothetical protein	
OYT1_00650	DM08DRAFT_01518	Ribonuclease P protein component	
OYT1_00667	DM08DRAFT_01792	Inosine-5'-monophosphate dehydrogenase	
OYT1_00668	DM08DRAFT_01791	hypothetical protein	
OYT1_00727	DM08DRAFT_01070	hypothetical protein	
OYT1_00729	DM08DRAFT_01072	hypothetical protein	
OYT1_00730	DM08DRAFT_01073	hypothetical protein	
OYT1_00741	DM08DRAFT_01083	hypothetical protein	
OYT1_00769	DM08DRAFT_02010	Cyclic di-GMP phosphodiesterase Gmr	
OYT1_00790	DM08DRAFT_02032	VanZ like family protein	
OYT1_00793	DM08DRAFT_02035	hypothetical protein	
OYT1_00794	DM08DRAFT_02036	hypothetical protein	
OYT1_00803	DM08DRAFT_01391	Flagellar L-ring protein precursor	
OYT1_00810	DM08DRAFT_01384	Flagella basal body P-ring formation protein FlgA precursor	
OYT1_00821	DM08DRAFT_00960	hypothetical protein	
OYT1_00836	DM08DRAFT_01382	Peptide methionine sulfoxide reductase MsrA 3	
OYT1_00837	DM08DRAFT_01381	hypothetical protein	
OYT1_00841	DM08DRAFT_01374	putative diguanylate cyclase YegE	
OYT1_00872	DM08DRAFT_02136	hypothetical protein	
OYT1_00889	DM08DRAFT_01321	Cyclic pyranopterin monophosphate synthase	
OYT1_00920	DM08DRAFT_00127	hypothetical protein	
OYT1_00924	DM08DRAFT_02159	Outer membrane protein P1 precursor	
OYT1_00933	DM08DRAFT_02151	Sensor histidine kinase LiaS	
OYT1_00937	DM08DRAFT_01691	hypothetical protein	
OYT1_00940	DM08DRAFT_01773	hypothetical protein	
OYT1_00958	DM08DRAFT_01755	FlgN protein	
OYT1_00966	DM08DRAFT_01743	SoxR reducing system protein RseC	
OYT1_00967	DM08DRAFT_01742	Sigma factor AlgU regulatory protein MucB precursor	
OYT1_00974	DM08DRAFT_01734	Endoglucanase precursor	bcsZ-like
OYT1_00975	DM08DRAFT_01733	Cyclic di-GMP-binding protein precursor	bcsB-like
OYT1_00976	DM08DRAFT_01732	Cellulose synthase catalytic subunit [UDP-forming]	bcsA-like
OYT1_00990	DM08DRAFT_01123	Flagellar assembly protein FliH	
OYT1_01042	DM08DRAFT_01221	hypothetical protein	
OYT1_01058	DM08DRAFT_01237	Outer membrane protein assembly factor BamC	
OYT1_01061	DM08DRAFT_01241	TLC ATP/ADP transporter	
OYT1_01066	DM08DRAFT_01245	Flagellar protein YcgR	
OYT1_01074	DM08DRAFT_01253	hypothetical protein	
OYT1_01075	DM08DRAFT_01254	hypothetical protein	
OYT1_01082	DM08DRAFT_02361	Xylulose kinase	
OYT1_01096	DM08DRAFT_02352	putative siderophore transport system ATP-binding protein YusV	
OYT1_01097	DM08DRAFT_02351	Hemin transport system permease protein HmuU	
OYT1_01100	DM08DRAFT_02348	bacteriophage N4 adsorption protein B	
OYT1_01108	DM08DRAFT_00968	hypothetical protein	
OYT1_01109	DM08DRAFT_00969	hypothetical protein	
OYT1_01117	DM08DRAFT_01005	hypothetical protein	

OYT1_01123	DM08DRAFT_01010	Sensor protein KdpD
OYT1_01134	DM08DRAFT_01022	Inner membrane protein alx
OYT1_01138	DM08DRAFT_00312	Secreted effector protein pipB2
OYT1_01146	DM08DRAFT_00907	hypothetical protein
OYT1_01153	DM08DRAFT_01512	Heme exporter protein D (CcmD)
OYT1_01166	DM08DRAFT_00117	Bacterial type II/III secretion system short domain protein
OYT1_01167	DM08DRAFT_00116	Murein DD-endopeptidase MepH precursor
OYT1_01168	DM08DRAFT_00115	hypothetical protein
OYT1_01173	DM08DRAFT_00209	Cyclic di-GMP phosphodiesterase Gmr
OYT1_01192	DM08DRAFT_00193	Transcriptional regulatory protein YehT
OYT1_01193	DM08DRAFT_00192	putative sensor-like histidine kinase
OYT1_01198	DM08DRAFT_00186	putative diguanylate cyclase YdaM
OYT1_01199	DM08DRAFT_00185	Vitamin B12-binding protein precursor
OYT1_01206	DM08DRAFT_00178	hypothetical protein
OYT1_01216	DM08DRAFT_01962	hypothetical protein
OYT1_01244	DM08DRAFT_00798	hypothetical protein
OYT1_01261	DM08DRAFT_00781	hypothetical protein
OYT1_01279	DM08DRAFT_01916	Phosphate-binding protein PstS precursor
OYT1_01299	DM08DRAFT_01899	hypothetical protein
OYT1_01303	DM08DRAFT_01895	Putative phospholipase A1 precursor
OYT1_01308	DM08DRAFT_01588	NADPH dehydrogenase
OYT1_01310	DM08DRAFT_01586	hypothetical protein
OYT1_01311	DM08DRAFT_01585	Aerotaxis receptor
OYT1_01317	DM08DRAFT_01579	Cobalt-zinc-cadmium resistance protein CzcC precursor
OYT1_01318	DM08DRAFT_01578	Cation efflux system protein CusB precursor
OYT1_01320	DM08DRAFT_01576	hypothetical protein
OYT1_01339	DM08DRAFT_01559	HDOD domain protein
OYT1_01349	DM08DRAFT_01214	Low conductance mechanosensitive channel YnaI
OYT1_01351	DM08DRAFT_00100	Signal transduction histidine-protein kinase/phosphatase DegS
OYT1_01445	DM08DRAFT_00024	Decarbamoylnovobiocin carbamoyltransferase
OYT1_01450	DM08DRAFT_00104	hypothetical protein
OYT1_01457	DM08DRAFT_01487	Transaldolase
OYT1_01458	DM08DRAFT_01488	Glucokinase
OYT1_01462	DM08DRAFT_01492	Putative oxidoreductasec/MT0587
OYT1_01469	DM08DRAFT_01499	hypothetical protein
OYT1_01474	DM08DRAFT_01828	HDOD domain protein
OYT1_01480	DM08DRAFT_01476	hypothetical protein
OYT1_01498	DM08DRAFT_01417	Bifunctional enzyme CysN/CysC
OYT1_01503	DM08DRAFT_02174	Type I restriction enzyme EcoKI M protein
OYT1_01507	DM08DRAFT_02169	Type I restriction enzyme EcoR124II R protein
OYT1_01517	DM08DRAFT_01747	Cyclic di-GMP phosphodiesterase Gmr
OYT1_01524	DM08DRAFT_01575	Cyclic di-GMP phosphodiesterase Gmr
OYT1_01531	DM08DRAFT_01859	Inner membrane metabolite transport protein YhjE
OYT1_01540	DM08DRAFT_01845	Thermostable hemolysin
OYT1_01543	DM08DRAFT_01842	Fatty acyl-CoA reductase
OYT1_01544	DM08DRAFT_01841	hypothetical protein
OYT1_01549	DM08DRAFT_01834	Putative universal stress protein
OYT1_01551	DM08DRAFT_01832	Tetratricopeptide repeat
OYT1_01554	DM08DRAFT_01686	Phage virion morphogenesis family protein
OYT1_01555	DM08DRAFT_01685	Phage Mu protein F like protein
OYT1_01557	DM08DRAFT_01683	hypothetical protein
OYT1_01558	DM08DRAFT_01682	hypothetical protein
OYT1_01560	DM08DRAFT_01680	hypothetical protein
OYT1_01561	DM08DRAFT_01679	hypothetical protein
OYT1_01563	DM08DRAFT_01675	hypothetical protein
OYT1_01564	DM08DRAFT_01674	N-acetyl-anhydromuranmyl-L-alanine amidase
OYT1_01572	DM08DRAFT_01665	Integrase core domain protein
OYT1_01573	DM08DRAFT_01664	hypothetical protein
OYT1_01574, OYT1_02670	DM08DRAFT_01663	hypothetical protein
OYT1_01577	DM08DRAFT_01660	hypothetical protein
OYT1_01578	DM08DRAFT_01659	hypothetical protein
OYT1_01581	DM08DRAFT_01657	hypothetical protein
OYT1_01582	DM08DRAFT_01656	hypothetical protein
OYT1_01584	DM08DRAFT_01655	Mu-like prophage I protein
OYT1_01585	DM08DRAFT_01654	Mu-like prophage major head subunit gpT
OYT1_01586	DM08DRAFT_01653	hypothetical protein
OYT1_01587	DM08DRAFT_01652	hypothetical protein
OYT1_01588	DM08DRAFT_01651	hypothetical protein
OYT1_01589	DM08DRAFT_01650	Phage tail sheath protein
OYT1_01590	DM08DRAFT_01649	Phage tail tube protein
OYT1_01595	DM08DRAFT_01646	hypothetical protein
OYT1_01596	DM08DRAFT_01645	Phage late control gene D protein (GPD)
OYT1_01597	DM08DRAFT_01644	Bacteriophage Mu Gp45 protein
OYT1_01598	DM08DRAFT_01643	Phage protein GP46
OYT1_01599	DM08DRAFT_01642	Baseplate J-like protein

OYT1_01600	DM08DRAFT_01641	hypothetical protein
OYT1_01638	DM08DRAFT_01351	putative diguanylate cyclase YdaM
OYT1_01645	DM08DRAFT_01359	cell division protein DedD
OYT1_01646	DM08DRAFT_01360	Colicin V production protein
OYT1_01680	DM08DRAFT_00435	Cytoskeleton protein RodZ
OYT1_01682	DM08DRAFT_00438	Pathogenicity locus
OYT1_01683	DM08DRAFT_00898	hypothetical protein
OYT1_01690	DM08DRAFT_00445	Phytochrome-like protein cph2
OYT1_01713	DM08DRAFT_01178	hypothetical protein
OYT1_01719	DM08DRAFT_01172	C4-dicarboxylic acid transporter DauA
OYT1_01724	DM08DRAFT_01158	hypothetical protein
OYT1_01737	DM08DRAFT_01146	Cyclic di-GMP phosphodiesterase Gmr
OYT1_01738	DM08DRAFT_01145	Cyclic di-GMP phosphodiesterase Gmr
OYT1_01777	DM08DRAFT_01137	Catechol-2,3-dioxygenase
OYT1_01794	DM08DRAFT_00402	hypothetical protein
OYT1_01824	DM08DRAFT_01098	hypothetical protein
OYT1_01825	DM08DRAFT_01097	Inner spore coat protein H
OYT1_01858	DM08DRAFT_00426	Sensor protein RstB
OYT1_01860	DM08DRAFT_00428	hypothetical protein
OYT1_01890	DM08DRAFT_02098	Outer membrane protein P5 precursor
OYT1_01899, OYT1_01974	DM08DRAFT_02111	hypothetical protein
OYT1_01900, OYT1_01975	DM08DRAFT_02112	hypothetical protein
OYT1_01903, OYT1_01977	DM08DRAFT_02114, DM08DRAFT_02190	hypothetical protein
OYT1_01904, OYT1_01978	DM08DRAFT_02115, DM08DRAFT_02189	hypothetical protein
OYT1_01907, OYT1_01981	DM08DRAFT_02118, DM08DRAFT_02185	hypothetical protein
OYT1_01909, OYT1_01983	DM08DRAFT_02120, DM08DRAFT_02183	hypothetical protein
OYT1_01910, OYT1_01984	DM08DRAFT_02182, DM08DRAFT_02121	hypothetical protein
OYT1_01911, OYT1_01985	DM08DRAFT_02122, DM08DRAFT_02181	hypothetical protein
OYT1_01912, OYT1_01986	DM08DRAFT_02180, DM08DRAFT_02123	hypothetical protein
OYT1_01913, OYT1_01987	DM08DRAFT_02179	DNA adenine methyltransferase YhdJ
OYT1_01920, OYT1_01995	DM08DRAFT_01596, DM08DRAFT_02233	Phage portal protein
OYT1_01924	DM08DRAFT_02229	hypothetical protein
OYT1_01926, OYT1_02001	DM08DRAFT_01602, DM08DRAFT_02227	Phage head-tail joining protein
OYT1_01941	DM08DRAFT_02213	hypothetical protein
OYT1_01969, OYT1_01894	DM08DRAFT_02199, DM08DRAFT_02106	hypothetical protein
OYT1_01970, OYT1_01895	DM08DRAFT_02198, DM08DRAFT_02107	DNA-invertase hin
OYT1_01971, OYT1_01896	DM08DRAFT_02197, DM08DRAFT_02108	hypothetical protein
OYT1_01972, OYT1_01897	DM08DRAFT_02196, DM08DRAFT_02109	hypothetical protein
OYT1_01979, OYT1_01905	DM08DRAFT_02188, DM08DRAFT_02116	hypothetical protein
OYT1_01980, OYT1_01906	DM08DRAFT_02186, DM08DRAFT_02117	hypothetical protein
OYT1_01982, OYT1_01908	DM08DRAFT_02184, DM08DRAFT_02119	hypothetical protein
OYT1_01989, OYT1_01914	DM08DRAFT_02178	hypothetical protein
OYT1_01990, OYT1_01915	DM08DRAFT_02177, DM08DRAFT_01591	hypothetical protein
OYT1_01991, OYT1_01916	DM08DRAFT_01592	hypothetical protein
OYT1_01992, OYT1_01917	DM08DRAFT_01593	HNH endonuclease
OYT1_01993, OYT1_01918	DM08DRAFT_01594, DM08DRAFT_02235	Phage terminase, small subunit
OYT1_01994, OYT1_01919	DM08DRAFT_01595, DM08DRAFT_02234	Phage Terminase
OYT1_01996, OYT1_01921	DM08DRAFT_01597, DM08DRAFT_02232	Putative signal peptide peptidase SppA
OYT1_01997, OYT1_01922	DM08DRAFT_01598, DM08DRAFT_02231	Phage capsid family protein

OYT1_01998, OYT1_01923	DM08DRAFT_01599, DM08DRAFT_02230	hypothetical protein
OYT1_01999	DM08DRAFT_01600	hypothetical protein
OYT1_02000, OYT1_01925	DM08DRAFT_01601, DM08DRAFT_02228	hypothetical protein
OYT1_02002, OYT1_01927	DM08DRAFT_02226, DM08DRAFT_01603	hypothetical protein
OYT1_02003, OYT1_01928	DM08DRAFT_02225, DM08DRAFT_01604	hypothetical protein
OYT1_02004, OYT1_01929	DM08DRAFT_01605, DM08DRAFT_02224	hypothetical protein
OYT1_02005, OYT1_01930	DM08DRAFT_01606, DM08DRAFT_02223	hypothetical protein
OYT1_02006, OYT1_01931	DM08DRAFT_01607, DM08DRAFT_02222	hypothetical protein
OYT1_02007, OYT1_01932	DM08DRAFT_01608	hypothetical protein
OYT1_02008, OYT1_01935	DM08DRAFT_01609, DM08DRAFT_02219	hypothetical protein
OYT1_02009, OYT1_01936	DM08DRAFT_01610, DM08DRAFT_02218	hypothetical protein
OYT1_02010, OYT1_01937	DM08DRAFT_01611, DM08DRAFT_02217	hypothetical protein
OYT1_02011, OYT1_01938	DM08DRAFT_01612, DM08DRAFT_02216	hypothetical protein
OYT1_02012, OYT1_01939	DM08DRAFT_01613, DM08DRAFT_02215	hypothetical protein
OYT1_02013, OYT1_01940	DM08DRAFT_01614, DM08DRAFT_02214	hypothetical protein
OYT1_02014	DM08DRAFT_01615	hypothetical protein
OYT1_02015, OYT1_01942	DM08DRAFT_01616, DM08DRAFT_02212	hypothetical protein
OYT1_02016, OYT1_01943	DM08DRAFT_01617, DM08DRAFT_02211	Peptidoglycan L-alanyl-D-glutamate endopeptidase CwlK precursor
OYT1_02017, OYT1_01944	DM08DRAFT_01618, DM08DRAFT_02210	hypothetical protein
OYT1_02018	DM08DRAFT_01619	Tetracycline resistance protein, class B
OYT1_02021	DM08DRAFT_01622	hypothetical protein
OYT1_02022	DM08DRAFT_01623	hypothetical protein
OYT1_02025	DM08DRAFT_01626	Cyclic di-GMP phosphodiesterase response regulator RpfG
OYT1_02026	DM08DRAFT_01627	hypothetical protein
OYT1_02027	DM08DRAFT_01628	hypothetical protein
OYT1_02028	DM08DRAFT_01629	hypothetical protein
OYT1_02029	DM08DRAFT_01630	Cation efflux system protein CusF precursor
OYT1_02030	DM08DRAFT_01631	hypothetical protein
OYT1_02037, OYT1_01902, OYT1_01976	DM08DRAFT_01637, DM08DRAFT_02113	hypothetical protein
OYT1_02078	DM08DRAFT_00921	murein peptide amidase A
OYT1_02088	DM08DRAFT_01921	Bacterial SH3 domain protein
OYT1_02092	DM08DRAFT_01925	O-Antigen ligase
OYT1_02105	DM08DRAFT_00707	hypothetical protein
OYT1_02114	DM08DRAFT_00715	hypothetical protein
OYT1_02121	DM08DRAFT_00759	putative diguanylate cyclase YdaM
OYT1_02122	DM08DRAFT_00758	Tetrathionate response regulatory protein TtrR
OYT1_02133	DM08DRAFT_00747	Histidine protein kinase DivJ
OYT1_02138	DM08DRAFT_00742	Lipopolysaccharide heptosyltransferase 1
OYT1_02156	DM08DRAFT_00728	hypothetical protein
OYT1_02165	DM08DRAFT_01295	Chaperone protein DnaK
OYT1_02173	DM08DRAFT_00704	hypothetical protein
OYT1_02174	DM08DRAFT_00703	hypothetical protein
OYT1_02186	DM08DRAFT_00691	DNA ligase
OYT1_02189	DM08DRAFT_00688	cell division protein FtsN
OYT1_02193	DM08DRAFT_00684	hypothetical protein
OYT1_02199	DM08DRAFT_00571	hypothetical protein
OYT1_02211	DM08DRAFT_00559	Fimbrial protein precursor
OYT1_02212	DM08DRAFT_00558	hypothetical protein
OYT1_02213	DM08DRAFT_00557	hypothetical protein
OYT1_02214	DM08DRAFT_00556	hypothetical protein
OYT1_02215	DM08DRAFT_00555	hypothetical protein
OYT1_02216	DM08DRAFT_00554	Neisseria PilC protein
OYT1_02219, OYT1_02054, OYT1_00595	DM08DRAFT_02322	Putative prophage phiR integrase
OYT1_02269	DM08DRAFT_00908	hypothetical protein
OYT1_02285	DM08DRAFT_00926	Multidrug export protein AcrF

OYT1_02286	DM08DRAFT_00925	Efflux pump periplasmic linker BepF
OYT1_02287	DM08DRAFT_00924	Fatty acid metabolism regulator protein
OYT1_02295	DM08DRAFT_02076	Outer-membrane lipoprotein LolB precursor
OYT1_02313	DM08DRAFT_02388	Cyclic di-GMP phosphodiesterase Gmr
OYT1_02320	DM08DRAFT_02394	Guanyl-specific ribonuclease Sa
OYT1_02345	DM08DRAFT_01804	hypothetical protein
OYT1_02352	DM08DRAFT_02296	hypothetical protein
OYT1_02358	DM08DRAFT_02301	diguanylate cyclase
OYT1_02360	DM08DRAFT_02303	Cyclic di-GMP phosphodiesterase Gmr
OYT1_02361	DM08DRAFT_02304	Signal transduction histidine-protein kinase BarA
OYT1_02363	DM08DRAFT_02305	Ankyrin repeats (3 copies)
OYT1_02377	DM08DRAFT_02312	hypothetical protein
OYT1_02384	DM08DRAFT_02320	AsmA family protein
OYT1_02398	DM08DRAFT_01110	putative voltage-gated ClC-type chloride channel ClcB
OYT1_02407	DM08DRAFT_01113	Periplasmic beta-glucosidase precursor
OYT1_02408	DM08DRAFT_01112	L-fucose-proton symporter
OYT1_02409	DM08DRAFT_01111	putative assembly protein
OYT1_02413	DM08DRAFT_02086	hypothetical protein
OYT1_02431	DM08DRAFT_01676	hypothetical protein
OYT1_02432	DM08DRAFT_01677	hypothetical protein
OYT1_02433, OYT1_01562	DM08DRAFT_01678	hypothetical protein
OYT1_02481	DM08DRAFT_01438	Cyclic di-GMP phosphodiesterase Gmr
OYT1_02482	DM08DRAFT_01437	hypothetical protein
OYT1_02484	DM08DRAFT_01436	hypothetical protein
OYT1_02486	DM08DRAFT_01435	Tyrosine-protein phosphatase YwqE
OYT1_02487	DM08DRAFT_01434	Tyrosine-protein kinase etk
OYT1_02488	DM08DRAFT_01433	Polysaccharide biosynthesis/export protein
OYT1_02489	DM08DRAFT_01432	hypothetical protein
OYT1_02490	DM08DRAFT_01431	O-Antigen ligase
OYT1_02535	DM08DRAFT_02053	hypothetical protein
OYT1_02536	DM08DRAFT_02054	NMT1/THI5 like protein
OYT1_02542	DM08DRAFT_02060	hypothetical protein
OYT1_02554	DM08DRAFT_01061	Rare lipoprotein A (RlpA)-like double-psi beta-barrel
OYT1_02556	DM08DRAFT_01993	cell division protein ZipA
OYT1_02565	DM08DRAFT_01981	Murein DD-endopeptidase MepH precursor
OYT1_02573, OYT1_01178	DM08DRAFT_00210	Phytochelatin synthase
OYT1_02575	DM08DRAFT_02400	Arabinose operon regulatory protein
OYT1_02577	DM08DRAFT_02398	3 beta-hydroxysteroid dehydrogenase/Delta 5-->4-isomerase
OYT1_02585	DM08DRAFT_01058	hypothetical protein
OYT1_02586	DM08DRAFT_01057	hypothetical protein
OYT1_02587	DM08DRAFT_01056	periplasmic repressor CpxP
OYT1_02624	DM08DRAFT_02396	hypothetical protein
OYT1_02625	DM08DRAFT_02397	hypothetical protein
OYT1_02686	DM08DRAFT_01715	Antitoxin MazE
OYT1_02687	DM08DRAFT_01714,	mRNA interferase MazF

* Annotation of OYT1 CDS by Prokka

Table S4. List of CDS of putative *c*-type cytochrome genes. Symbols: +, found in the top 10 blast hits; -, not found in the top 10 blast hits.

OYT1 tag#	R-1 tag#	Annotation*	Sideroxydans ES-1	Gallionella ES-2	Mariprofundus PV-1, M34, or EKF-M39	Subcellular localization**	Note
OYT1_00080	DM08DRAFT_02264	Cytochrome c_6	+	+	+	Periplasmic	ActE-like
OYT1_00085	DM08DRAFT_02259	Class III cytochrome *c* family protein	+	+	+	Periplasmic	ActA-like
OYT1_00185, OYT1_00186	DM08DRAFT_00329	Cytochrome c_{551}	+	+	-	Periplasmic	
OYT1_00427	DM08DRAFT_00513	*cbb*$_3$-type cytochrome *c* oxidase subunit FixP	+	+	+	Unknown	
OYT1_00429	DM08DRAFT_00515	Cytochrome *c* oxidase, mono-heme subunit/FixO	+	+	+	Cytoplasmic	
OYT1_00490	DM08DRAFT_00581	Cytochrome c_1	+	-	+	Non-cytoplasmic	
OYT1_00493	DM08DRAFT_00852	Planctomycete cytochrome *c*	+	-	-	Non-cytoplasmic	
OYT1_00754	-	Cytochrome c_{552}	-	+	-	Periplasmic	
OYT1_00909	-	hypothetical protein	-	-	+	Periplasmic	
OYT1_01615	-	Cytochrome *c*'	-	+	-	Non-cytoplasmic	
OYT1_01744	DM08DRAFT_01138	Cytochrome c_{552}	+	+	-	Non-cytoplasmic	
OYT1_01963	-	Cytochrome *c*'	-	+	-	Non-cytoplasmic	
OYT1_02245	DM08DRAFT_00859	Cytochrome c_{551}	+	+	-	Periplasmic	
OYT1_02326	DM08DRAFT_01280	Cytochrome c_{551}	-	+	-	Periplasmic	
OYT1_02356	DM08DRAFT_02299	hypothetical protein	+	+	-	Non-cytoplasmic	

* Annotated by Prokka for OYT1 CDS; ** predicted by PSORTb
